# The Exchange Breathing Method for Seizure Intervention: A Historical and Scientific Review of Epilepsy and Its Evolving Therapeutic Paradigms

**DOI:** 10.3390/jpm15080385

**Published:** 2025-08-18

**Authors:** Frederick Robert Carrick, Pamela Daniels, Stephen Pelletier, Sofia Prysmakova, Ahmed Hankir, Mahera Abdulrahman, Nouf Al-Rumaihi

**Affiliations:** 1 College of Medicine, University of Central Florida, Orlando, FL 32827, USA; 2 Centre for Mental Health Research in Association with University of Cambridge, Cambridge CB2 1TN, UK; ahankir@uwo.ca; 3 Burnett School of Biomedical Science, University of Central Florida, Orlando, FL 32827, USA; 4 MGH Institute of Health Professions, Boston, MA 02129, USA; 5 Department of Neurology, Carrick Institute, Cape Canaveral, FL 32920, USA; pdaniels@faculty.carrickinstitute.com (P.D.); sprysmakovarivera@fgcu.edu (S.P.); n.alrumaihi@scfhs.org.sa (N.A.-R.); 6 Saudi Commission for Health Specialties, Riyadh 11614, Saudi Arabia; 7 Harvard Medical School, Boston, MA 02115, USA; stephen_pelletier@hms.harvard.edu; 8 Department of Political Science & Public Administration, Florida Gulf Coast University, Fort Myers, FL 33965, USA; 9 School of Public Administration, University of Central Florida, Orlando, FL 32816, USA; 10 School of Medicine, Cardiff University, Cardiff CF14 4YS, UK; 11 Schulich School of Medicine and Dentistry, University of Western Ontario, London, ON N6A 5C1, Canada; 12 Department of Informatics and Smart Health, Dubai Health Authority, Dubai 43111, United Arab Emirates; marad@dha.gov.ae; 13 Department of Public Health, Mohammed Bin Rashid School of Medicine, Dubai 88905, United Arab Emirates

**Keywords:** epilepsy, seizure, brain function, respiration, exchange breathing method, nasal anatomy

## Abstract

Epilepsy is a complex and ancient neurological disorder affecting approximately 50 million individuals globally. Despite significant advancements in pharmacological treatments, surgical procedures, and neurostimulation techniques, a substantial subset of patients remains pharmacoresistant or experiences intolerable side effects, highlighting the need for novel, safe, and effective interventions. In this review, we examine a promising non-invasive technique known as the Exchange Breathing Method (EBM), developed through the observations of Gemma Herbertson, a British mother who discovered that exhaling gently into her son’s nostrils could consistently interrupt ongoing seizures. The EBM has since gained anecdotal support from a growing international community reporting similar positive outcomes. This paper situates the EBM within the broader historical and clinical context of epilepsy treatment, tracing its evolution from ancient practices to modern therapeutic strategies. We explore the neurophysiological mechanisms that may underlie the EBM, particularly its interaction with autonomic and respiratory pathways implicated in seizure modulation. By integrating emerging grassroots data with current scientific knowledge, this review proposes a rationale for further empirical investigation into the EBM and its potential role in the personalized, emergency management of epilepsy.

## 1. Introduction

Epilepsy is one of the most ancient and enigmatic neurological disorders, affecting approximately 50 million people worldwide. Characterized by recurrent, unprovoked seizures, epilepsy encompasses a broad spectrum of manifestations ranging from brief lapses in awareness to full-body convulsions and presents significant challenges in diagnosis, treatment, and long-term management. Despite advances in pharmacological therapy, surgical interventions, and neurostimulation techniques, a considerable percentage of patients remain pharmacoresistant, and many treatments carry substantial side effects or limitations. As such, the search for alternative, safe, and effective seizure interventions remains a critical pursuit in modern neuroscience and medicine.

In this context, a novel, non-invasive method has emerged from an unexpected source: the observations of a British mother, Gemma Herbertson. Herbertson, whose young son suffered up to 30 seizures per day, discovered that she could immediately stop his seizures by breathing into his nose—covering his nostrils with her mouth and gently blowing her breath into his nostrils. Remarkably, this simple act of respiratory intervention appeared to consistently interrupt the seizure activity. Naming her technique the Exchange Breathing Method (EBM), Herbertson began sharing her discovery informally with friends, relatives, and eventually a growing international community. Today, her private support and testimonial group comprises thousands of users across diverse geographic and clinical backgrounds, many of whom report similar positive outcomes with the EBM.

This paper aims to explore the Exchange Breathing Method within the broader framework of epilepsy research and treatment. To do so, we will first examine the clinical and epidemiological profile of epilepsy, including its neurological underpinnings and classifications. We will then survey the historical evolution of epilepsy treatment from early spiritual and herbal remedies to contemporary neuropharmacology and neurosurgery, highlighting both the progress made and the therapeutic gaps that persist. Against this backdrop, we introduce and evaluate the EBM as a potential adjunct or alternative to existing modalities. By synthesizing user-reported outcomes with current scientific understanding of seizure physiology and autonomic respiratory mechanisms, this review seeks to assess the plausibility and therapeutic potential of the EBM and to consider how this grassroots innovation may inspire new directions in the treatment of epilepsy.

## 2. Epilepsy—Definition, History, and Incidence

Epilepsy is one of the most common and taxing neurological disorders, routinely manifesting outwardly as seizure activity stemming from paroxysmal yet persistent disordered neurological function in the brain [1]. The epileptic seizure activity can be physically characterized as jerking, trembling, spasmodic, or clonic types of body movements that vary in intensity and frequency in the person affected [2]. Yet, not all persons with seizure activity have epilepsy [3], and not all seizure activity is outwardly convulsive or tangible [1]. However, all seizure activity, regardless of quality, is a symptom of abnormal brain activity. Therefore, epilepsy is classified as a disease and hence recognized in its entirety by a grouping of signs and symptoms [4]. Seizure activity can occur and is common as the result of other diseases (primary diagnosis) such as neurological and genetic syndromes [5], Alzheimer’s [6], TBI [7], neurodegeneration [8], tumors [9], and stroke [10], or from acute metabolic, toxic, and infectious events [3]. This is by no means an exhaustive list of all the conditions that can provoke seizure activity (epileptogenesis). Still, it should highlight that seizures can and do result from a wide range of neurological causes and consequently have large-scale impacts on society.

The distinction between seizure and epilepsy is confusing and has been bogged down by the prevailing scientific, religious, and biased opinions of historical times [4]. Derived from Greek, the word seizure translates to “to take hold,” and reports describing seizures and epilepsy date as far back as the Paleolithic period [4]. Perhaps contributing to this misunderstanding is that epileptic activity, or seizure activity, is frequently the first noticeable and vigorous symptom observed [4], resulting in frequent interchangeable term usage, thus playing a role in the unclear demarcation between the two throughout history. The development of various classification systems has been instrumental in clearly defining this terminology, thereby enhancing accurate assertions about the underlying pathophysiology and supporting patient and healthcare administrators’ understanding and cooperation [4]. However, this has also been fraught with challenges and criticisms. Even within the classification systems, such as the ILAE of 2017, there are three equivocal descriptive categories for epilepsy and seizure types [11]. Please review [11] for a critical view of the 2017 ILAE classification system for epilepsy. Also, please see [4] for an extensive and enlightening historical and evolutionary account of the distinctions and similarities between the terminology of seizure and epilepsy. We recognize they are not entirely the same entity: seizure is the symptom, and epilepsy is the disease. However, the following discussion is applicable and relevant to the effects of both on the physical, mental, emotional, financial, physiological, investigative, and therapeutic aspects and impacts.

Epilepsy is a heterogeneous disorder (disease) that can be recognized by a grouping of signs and symptoms that traverses a wide range of age groups, primary diagnoses, genders, ethnicities [12], and socioeconomic and geographical landscapes. Therefore, it is no surprise that its etiology is recognized as multifactorial and is still under investigation. However, advancements in animal modeling and the culturing of human stem cells and cerebral organoids have led to a greater understanding of the intricate and elusive pathophysiological causes of epilepsy and, hence, a synergistic goal for improved treatments for all types of epilepsy [1]. Contributing to this understanding are the roundworm (*C. elegans*), Drosophila, zebrafish, rat, and mouse, all of which have contributed to human disease modeling, and epilepsy is no exception. An estimated 75–80% of human disease genes have genetic orthologues in Drosophila, zebrafish, rat, and *C. elegans*, with additional conserved neurotransmitters, ion channels, and nervous system organization across species [1]. Thus, these animal kindling models (sensitization of brain circuitry) have become invaluable for understanding seizures, epilepsy, and pharmacological applications [13]. The above models all have advantages and disadvantages regarding the reproducibility, validity, and accuracy of research investigations and conclusions into this complex disorder, and each must be considered appropriately. For example, network dysfunction can be expressed as too much or too little neuronal function, and this is determined, in part, by whether a genetic mutation is a missense, nonsense, or deleted mutation [1]. Please see [1] for a detailed outline review of the above reflections.

Although there are many aspects to consider in epilepsy, there is broad agreement that it is a syndrome that can be siloed into six principal etiological categories: Structural, Genetic, Infectious, Metabolic, Immune, and Unknown [14]. Regardless of the etiology, all cases result in some form of hyperexcitable, erroneous, and dysynchronous neurological network activity in the brain, with seizures as their core manifestations [1], subsequently causing significant aberrant changes in the brain’s neuronal networks [2]. The basis for this persistent hyperexcitability lies in the progressive structural and chemical neurotransmission and neuronal modifications, including epigenetic alterations [15] that can begin within minutes of the first seizure [16]. More specifically, genetic causes are thought to be related to differential expression and “complex-inheritance and single-gene mutations on susceptible alleles” [15,17]. There are more than a hundred single genes known to cause epilepsy, some sharing a commonality with other neurodevelopmental disorders of the brain, like autism [1], and more than 1000 associated genes that form the pathological genetic basis of Epilepsy [2]. With the advent of advanced genomic testing, trio sequencing [1], AI, “next-generation testing (NGS),” MRI technological advancements (7T), and increased reporting and recognition, along with progressive, comprehensive, and updated classification systems, the “concept of the etiology of epilepsy” will continue to evolve [18]. Please see [18] for a thorough and “pragmatic” review of contemporary epilepsy classifications.

Epilepsy is associated with substantial costs, cognitive and psychological comorbidities, and stigma, including discrimination and misconceptions [2]. It is thus recognized by the World Health Organization (WHO) as a significant global health burden [2,19]. People who have epilepsy are eight times more likely to experience migraine, depression, cardiorespiratory diseases, autoimmune diseases, and other physical and neuropsychiatric conditions [18]. Even family members of people with epilepsy (PWE) are at significant risk for developing clinical depression [20]. With epilepsy alone accounting for nearly 1% of the global disease burden [21], accurate classification of epilepsy is essential to help clinicians, providers, family members, caregivers, and patients appreciate and navigate the totality of the disease burden in everyday life [18]. Epilepsy can affect all ages. However, the highest incidence is generally reported in the extreme demographic age ranges, resulting from multifactorial origins [1]. For example, in the very young, the brain is fragile and vulnerable to many insults, and incidence reporting in infants under one has increased due to enhanced medical technologies increasing survivability and prominent symptomology presentation [18]. In older adults 65 and older, the incidence rate rises to 240 per 100,000 [22] due to elevated cases of cardiovascular disease, neurodegeneration, tumors, and traumatic brain injuries [1,18]. In both age groups, the incidence trend is higher in biological males [23].

Complete agreement and lucidity are lacking on the true incidence and prevalence of epilepsy, in part due to inaccuracy and variances in reporting, geographic, sociodemographic, and ethnic profiles; however, based on a meta-analysis and systematic review of international studies, the prevalence of “active epilepsy was reported to be 6.38 per 1000 persons with an annual cumulative incidence of 67.77 per 100,000 persons” [19]. Childhood epilepsy (age less than 60 months) has also had mixed incidence rates reported. However, a population-based prospective study by Hunter et al. found similar rates as previously reported at 61.7 per 100,000 children [24]. They also concluded that almost two-thirds of the cases were of unknown etiology [24]; this is consistent with Anwar et al.’s reporting that “40 out of 100 cases of epilepsy are known” [2]. Hunter et al. also reported that 19% were genetic in origin and 24% were structural, and, unlike adults, low socioeconomic status was not a factor [24].

Accurate recognition and reporting of childhood epilepsy, particularly when presenting with a “first seizure,” is essential in preventing erroneous and potentially deleterious reporting, incorrect treatment, and stigma associated with the syndrome [25]. In 1993 [26], the International League Against Epilepsy (ILAE) determined that a diagnosis of epilepsy by clinicians and in epidemiologic studies must require two unprovoked seizures that occurred at least 24 h apart [25]. Yet, in 2014, the working definition was updated, allowing for one unprovoked seizure if the person is believed to have at least a 60% risk of reoccurrence within the next 10 years [25]. See [25] for the defined diagnostic risk criteria. Furthermore, the ILAE, in 2017, published a position paper on epilepsy classifications based on current scientific developments and extensive feedback from the epilepsy community [14]. This multilevel classification system is an essential guide for patients and clinicians [14]. In summary, this multitiered approach includes seizure type (focal, generalized, and unknown onset), epilepsy type (based on the previously mentioned 2014 definition), and finally, epilepsy syndrome, inclusive of several imaging results (EEG profiles) [27] and seizure types with characteristic comorbidities [14]. The latter category is perhaps the most relevant for this discussion as it is associated with specific etiological, age, and treatment paradigms [14].

A systematic approach and caution are prudent when considering a diagnosis of childhood epilepsy, as there are several potential mimics. A careful and detailed history and examination, along with diagnostic testing, such as an electroencephalogram (EEG) and MRI, can elucidate a potential epileptic mimic such as benign sleep myoclonus (hypnagogic jerks), self-gratification phenomena, non-epileptic psychogenic seizures, parasomnias, hallucinations, trauma-induced non-epileptiform seizures, breath holding, and reflex anoxic seizures from an accurate diagnosis of epilepsy [28]. Moreover, Syncope, the most common mimic [25], Benign Positional Vertigo (BPPV), Tics, Sandifer Syndrome, and Paroxysmal Kinesigenic Choreoathetosis (PKC) can mimic seizure activity in infants, toddlers, and young children, leading to misdiagnosis and unsuitable treatment [29]. Strong seizure semiology knowledge is crucial and assists considerably in correctly identifying and classifying epilepsy and its mimics [2]. Unfortunately, no non-ictal readily accessible biomarker exists to determine an ensuing epileptogenic event reliably [2].

Furthermore, seizures are the most common pediatric emergency for a neurological event, accounting for 1% of all visits to emergency rooms and 4–10% of childhood visits with diverse etiological and presentational profiles [30]. Febrile seizures, the most common type, occur in 2–5% of children aged six months to five years, with equivocal occurrence rates among genders [31], making these frequent “first seizure” attacks [30]. Initially considered benign with no future consequences or progression, they are now being viewed differently, despite being a single event. The suggestion is that the occurrence of febrile seizures may indicate that the brain is already constitutively different from the brain that does not respond to an insult with fever [32]. Currently, there are not enough studies reporting long-term consequences, such as cognitive, behavioral, mortality, and psychiatric complication rates; a few longer-term studies, such as [33,34], report most patients with no concurrent neurological abnormality do not advance into epilepsy, even after childhood status epilepticus [33] but that long-term adverse diagnoses and outcomes are associated with unfavorable underlying neurological status and that the outcomes are based on the initial cause of the seizure [33]. Yet, Yoong et al. reported a demonstrable hippocampal loss in children with repeated status epilepticus that was not associated with etiology but rather with the number of previous seizure insults [34]. In contrast, Martinos et al. found that etiology is the most significant predictor of long-term cognitive outcomes at 10 years [35]. Please review [31,32,33,34,35] for a broader understanding, as this subject, like epilepsy itself, is complex and much is still to be learned.

Worldwide, over 68 million people are affected by the devastating effects of epilepsy and its persistent consequences [36]. Although there are continued and substantial investigational efforts to understand the pathophysiology of epilepsy, it remains elusive. In both animal and human investigations, “over 60 neuronal subtypes” in specific and singular cortical and hippocampal regions with varying influences are suggested to be causative in seizure production [36]. Yet, in others, there are reductions in “somatostatin and neuropeptide Y positive GABAergic interneurons” in critical hippocampal regions, leading to Temporal Lobe Epilepsy (TLE) [36]. Exactly how these individual neuronal subtypes affect epileptogenesis and how the epileptic events subsequently affect those neurons is undetermined [36]. However, recently, the use of single-cell transcriptomics revealed thousands of dysregulated genes across all neuronal subtypes in TLE, with a preponderance affecting the GABAergic interneurons and glutamate-signaling genes exhibiting varying levels of severity, and that these alterations resided primarily in neuronal modules or clusters belonging to a shared network, exposing “layer-wise dysregulation” [36].

## 3. Sudden Death in Epilepsy (SUDEP)

Sudden death in epilepsy (SUDEP) is estimated to occur in one in every 1000 persons diagnosed with epilepsy; however, the rates are potentially higher (2.3–1000), as it seems to be under-recognized, carries ascertainment bias, and the identification depends on the research study populations and conditions [1,37]. In medically intractable seizures, SUDEP is the most common cause of death and accounts for one-third of deaths in pediatric patients with epilepsy (PWE) [38]. It accounts for 18% of deaths in the general population of PWE [39,40]. Among neurological diseases, SUDEP is only second to stroke for “years of potential life lost” [37,41]. Over a 40-year trajectory [42], pediatric patients were found to have an increased 7% cumulative risk for SUDEP, totaling a 38% death rate [43]. SUDEP, principally, is defined as death that occurs during or after a seizure, is unexpected, has no other known cause, and is non-traumatic, non-drowning, and unpredictable [37]. Additionally, it is frequently unwitnessed and has a high incidence during sleep in PWE [1,37]. The most significant predictor reported was the existence of generalized tonic–clonic seizures (GTCSs), leading to a 15 times higher mortality rate in persons with three or more seizures per month. Yet, the occurrence of SUDEP can also occur in typical epilepsy syndromes, leading to unclear comprehension of the fundamental pathophysiological mechanisms underlying SUDEP [38].

It is recognized that genetic mutations in SCN1A and SCN8A play a role in documented cases of SUDEP and PWE, albeit with different physiological consequences [37]. For an older but good outline of underlying causes, see [40,44]. Likewise, other mechanisms have been suggested, such as autonomic dysfunction, brainstem depolarization causing respiratory arrest [45], and cardiac arrhythmias [46]. The prevailing historical pathophysiological mechanism behind SUDEP has been that it is primarily cardiac as a result of the vigorous stress of the seizure episode; however, over the past few decades, the ability to monitor PWE in epilepsy-monitoring units (EMUs) points to changes in breathing, such as inhibition of the respiratory rate and central apnea [38]. This is not unanticipated, as evidence of respiratory distress was recognized by John Hughlings Jackson in the late 19th century [47,48]. In 2013, a seminal publication by Ryvlin et al. called the MORTEMUS study observed terminal central apnea occurring before cardiac asystole and deemed it the initiating source [49] More recent studies have suggested that post-central apnea could be a possible biomarker for SUDEP [50].

In line with those mentioned earlier, a multimodal study involving 20 epileptic patients by Harmata et al. consistently found that ictal apnea preceded postictal apnea when apnea occurred, concluding that there is a shared physiological mechanism underlying both or that one is a severe continuation of the other [51]. These findings were found in both focal and generalized convulsive seizures, possibly pointing to a disruption of the brainstem respiratory network [51]. Specifically, a small focal region within the amygdala (pAIR site) causes apnea that can persist for minutes after stimulation [51]. The authors noted a reduction in BOLD activity in the pontomedullary respiratory rhythm neurons, along with a lack of air hunger and absence of emotional responses to air hunger, and the subjects were unaware themselves that they had stopped breathing, even with rising CO_2_ levels post-amygdala stimulation [51]. A possible mechanism for inhibiting the CO_2_ reflex and air hunger awareness (interoception) can be explained by the anatomical connections between the amygdala and insula. Activity in the ventral insula increased after amygdala activation and is theorized by the authors to represent dysfunctional neural circuitry (inhibitory neuronal upregulation), which inhibits the normal insular identification and subsequent respiratory adjustments in response to climbing levels of CO_2_ [51]. Direct projections from the insula to the ventral pontomedullary region and NTS are reported, as well as indirect projections to the NTS from the fastigial nucleus of the cerebellum [52]. These connections lend support and establish that the insula and the NTS are significantly involved in many autonomic and visceral functions, particularly in various aspects of the respiratory network.

Additional studies have also reported neurologically persistent and unrecognized apnea. In a small pediatric continuous monitoring study by Rhone et al., while observing apneic seizures with corresponding seizure spread to the amygdala, despite abnormal breathing postictally, the children did not experience dyspnea, shortness of breath, or any signs of emotional respiratory distress [38]. Again, concernedly, the children were completely unaware of their hypoventilatory state. Yet, not all PWE may be prone to postictal apnea, possibly due to patient and seizure heterogeneity, and some may have a hemispheric laterality of susceptibility. Support for dysfunctional amygdala laterality is seen in Schön and colleagues’ work, which concluded that the right insular cortex was associated with less perception of breathlessness [53]. When considering the patient and seizure heterogeneity, we can look at Salami et al.’s work using cross-frequency coupling [54]. They analyzed 378 seizures from 43 patients. They noted different seizure patterns developing from the same regions within the same patient and between patients with similarly identified pathologic sites, thus, in part, highlighting the nuanced nature of neuronal network activity occurring during and after a seizure [54]. Furthermore, they noted variations in hormonal status, medication usage, duration of epilepsy, sleep-wake cycles, and stress to be confounders and influencers to pattern recordings [54]. Adding to this are the dynamic, rapidly changing, and potentially distinct intracellular and extracellular metabolic [55], vascular [42], and neuronal patterns that exist within each PWE [54]. Interestingly, 116 and 111 seizures recorded in Salami’s work were in the hippocampus and lateral temporal regions, respectively [54]; this is significant because, as reported by Burman et al., the hippocampus is a “favored region” in seizure activity because of its predisposition for self-perpetuating intrinsic network hyperexcitability and vulnerability to insult [55].

Relatedly, postictal focal hypoperfusion resulting from local vasoconstriction regional to the seizure and its tangential propagation is identified and deemed the neurovascular hypothesis [39,56,57]. George et al. observed in their mouse model both the NTS and the preBötzinger Complex became severely hypoxic, with _p_O_2_ dropping below 4 mmHg just before apnea occurred [39]. Anything below 10 mmHg is considered injurious and severely hypoxic to the brain [57]. In a rat model by Farrell et al., _p_O_2_ levels dropped below 10 mmHg for an extended period even after a brief seizure and proposed a vascular component (neurovascular hypothesis) in addition to the accepted electrical dysfunction mechanism underlying seizure behaviors and comorbidities like SUDEP [56]. In this study, Farrell’s group extended their hypoperfusion model from animal to human clinical epilepsy using MRI within 60 min of seizure activity [57]. Their theory and findings point to the observed hypoxia and restricted perfusion resulting from COX-2 enzymatic postsynaptic activity on vessel diameter with perpetuating downstream effects of _L-_type calcium channels, both fostering a systematic vasoconstriction state [56]. Intriguingly, forebrain and hippocampal seizures are suggested to disseminate to the lower respiratory brainstem structures, inducing vasoconstriction postictally and ending in severe hypoxia and hypercapnia systemically [56,57]. Please review [56,57] for added depth and explanation of the physiological mechanisms studied. Please see below, and see [37,38,41,51,58] for discussions on postictal apnea, the amygdala’s role, and unifying aspects behind SUDEP. Please review [52] for research on insular projections involved with visceral functions and [42,56,57] for more on the emerging neurovascular theory involved with seizures.

## 4. Treatments

As mentioned, a complete pathogenic picture is still lacking for seizures and epilepsy. However, it is recognized to involve highly complex biological processes, genetic variations, and neuronal developmental and acquired pathophysiological properties that are multifaceted and challenging to understand fully [59]. Considering the currently known biological pathways affected in epileptogenesis mainly due to molecular biological studies, a clearer understanding of the presently accepted therapeutic interventions can be obtained. See [55] for more insight. In general, the biological organization can be summarized into four groupings; genes that control membrane ion channels, enzyme regulator genes, transporters, and other essential cellular functions, such as cell adhesion, are recognized as central biological pathways involved in Epilepsy [2]. One of the primary treatment goals for all types of seizures is symptomatic relief, as recurrent and refractory seizures inflict a significant burden on the patients, families, caregivers, and, eventually, society as a whole [59]. Evidence exists that 85% of pediatric epilepsies are neuropsychologically impaired before surgery, highlighting that the neurological, cognitive, comorbidity, and psychosocial effects can be tremendous [59]. Clinicians must be familiar with a breadth of contemporary antiseizure therapies so that they might understand options and compare them with the EBM.

Antiepileptic Drugs (AEDs), now referred to as antiseizure medications (ASMs), are considered a mainstay and first line of defense in the treatment of seizures [59,60]. Yet, 30% to 50% of seizures are reported to be refractory to drug therapy, including multi-drug therapy [59,60,61]. Bromide was the first ASM identified in the 18th century for treating seizures [62]. In 1912, a German physician discovered phenobarbitone because of its sedating effects. It was this sedating effect of phenobarbitone that led to investigations for nonsedating compounds like Phenytoin in 1938, followed by Carbamazepine in 1953, which became the most prescribed ASM worldwide [62]. The discovery of valproate rolled into the discovery of Benzodiazepines, and through the next five decades, other medications, such as levetiracetam, Lamotrigine, and Perampanel, were investigated and utilized [62]. We are now in the era of third-generation ASMs. These drugs are approved for add-on therapies in adult patients [60]. The tolerability and efficacy of all ASMs are still challenging and inadequate. Please see [60] for a systematic review and metanalyses on third-generation ASMs.

The repertoire of ASMs in use is based on the phenotypic variances found in ion channel and ion channel transporter dysfunction, hormone and neurotransmitter alterations, and changes in calcium-activated kinases and vesicle release proteins [63]. For example, valproic acid, various carbonic anhydrases, and levetiracetam are theorized to reduce brain pH by altering the functionality of ion channels and cellular enzymes [64]. Levetiracetam, a second-generation ASM approved over 20 years ago, affects pH through complex mechanisms primarily at the synaptic vesicle [65]. Please review [65,66,67] for more discussion on ASMs’ mechanisms of action.

Historically, ASMs have been administered through various routes, such as oral administration, IV, and rectal. Yet, in emergency seizure situations and drug-resistant epilepsy (DRE), these routes may not be feasible or effective as solo treatments. Additionally, regarding IV routes of administration, patent and accessible blood vessels are required. Given the increased incidence of seizures in children and older adults, such as in status epilepticus (SE), other options are needed as they tend to have more narrow or less accessible blood vessels for reliable and secure injections [68]. Consequently, investigations into other rescue applications are emerging. Buccal, intramuscular, subcutaneous, interpulmonary, and more recently, nasal administration of various drugs, like benzodiazepine, have been approved by the FDA as rescue therapy in acute repetitive seizures (seizure clusters) [69,70].

Specifically, the extensive and superficial intranasal vascular supply and neurological anatomical substrates create a leading area for “direct-to-brain and systemic delivery” of medications for immediate therapeutic effects of seizure rescue [70]. Not only is the intranasal blood supply superficially extensive, but the superiorly placed olfactory sensory neurons (OSNs) and the widespread trigeminal nerve innervations all provide a direct path with limited barriers into the CNS via the olfactory bulb and cerebrospinal fluid [71]. Intranasal dosing is quick, convenient, non-invasive, and potentially self-administered, reduced dosing volume for expedited and positive therapeutic effects [71]. Nose-to-brain (NTB) applications allow for direct and indirect entry into the brain parenchyma through the blood–brain barrier (BBB) via systemic circulation (indirect) and directly by the OSNs and TSNs [68]. Animal studies are demonstrating that direct NTB application of drugs like Diazepam occurs from uptake in the OSNs, as well as the TGN and CSF [68]. Watanabe et al. suggest that the TGN, not the OB or the CSF, has a more significant direct effect on the brain, especially in regions like the thalamus [68]. Other studies utilizing insulin and caffeine concur regarding direct delivery via the TGN, olfactory bulb, and CSF, see [68]. Please review [69,70,72,73,74,75,76] for an in-depth review of anatomy, physiology, safety, effectiveness, and challenges concerning upcoming rescue therapies.

Surgical interventions are generally considered when there is an observable epileptogenic focus that can be identified when other therapies fail, with DRE, and when the benefit-to-risk ratio is acceptable [77]. Victor Horsley, in 1886, pioneered surgical intervention when he operated on a young man who had developed epilepsy resulting from a head injury [77]. Overall success rates have increased but can vary widely. A 2019 surgery review by Cochrane Library has identified several factors that may increase the success of the surgical outcome, for example, a well-described area of abnormality on imaging associated with EEG results and ictal semiology, scarring due to childhood febrile seizures, right-sided resection, and hippocampal removal [77]. Helmstaedter et al., in a large surgical cohort, also identified younger age, fewer ASMs, and later onset of epilepsy, along with better cognitive baseline performance, led to enhanced surgical outcomes in all domains [78]. As this topic is complex and not the focus of this paper, we urge the reader to review [77] for an extensive review of the literature covering surgical interventions and their implications for clinical practice.

Dietary interventions have reduced seizure activity and epilepsy since the early 1920s [79]. Specifically, in 1921, the Mayo Clinic in Minnesota ventured into the ketogenic diet (KD) for refractory epilepsy in children due to the immature and incomplete status of drug therapy available [79]. As drug therapies evolved, the KD treatment drastically declined. However, with the creation of the Charlie Foundation, a parent support group for children with epilepsies, the John Hopkins Hospital initiated a resurgence of this treatment modality around 1997 [79]. Presently, there are four recognized diets for the treatment of epilepsy: the original ketogenic diet (KD), the medium-chain triglyceride diet (MCT), the modified Atkins diet (MAD), and, finally, the glycemic index diet [79]. While these diets have been used for decades, the mechanism of action is still not entirely understood. While clinical improvement can be seen in days [80], it is recommended that these diets are maintained for a minimum of three to six months, with two years as the ultimate goal [79]. Some side effects reported are hypercholesteremia, lack of adherence, mineral deficiencies, unintended weight loss, and digestive complaints [79]. These may be ameliorated with adjunctive therapies like mineral supplementation and modification of initial implementation. Typically, these diets are utilized in young children, adolescents, and adults, likely due to adherence and safety concerns. However, in a 2020 systematic review of adolescents’ and infants’ success, responder rates were like those of adults. In summary, the authors reported that 57.4% of infants with infantile spasms experienced a seizure reduction greater than 50% within six months of implementation. In children two years and older, RRs were 38–56% and had at least 50% seizure reduction. Please see [80] for an attractive and novel thorough review of the KD mechanism of action in infantile seizures.

Various types of neurostimulation are currently used to reduce seizure activity. Since the early 1920s, interest and observations have existed on the effect of autonomic and cranial nerve activity on spontaneous movements, blood pressure, and seizure activity [81]. However, it was the seminal work by Moruzzi and Magoun in 1949 that confirmed the direct stimulation of the ascending reticular formation, abolishing synchronized discharges in the brainstem and having unmediated cephalic impacts, which greatly influenced future investigations in this arena [82]. As a result, others who followed this work have postulated that stimulation of widespread, non-somatotopic cranial nerves, such as cranial nerves 5, 9, and 10, may cause desynchronization of cortical activity in a hypersynchronized state, as seen in intractable seizures [83]. The effect of Vagus nerve (CN 10) stimulation took the main stage in the decades that followed. Based on the evidence provided by both animal and human epileptic models, Vagal nerve stimulation (VNS) was approved by the FDA in 1997 as an adjunct therapy to focal-onset seizures and soon became a standard treatment for seizures in persons with intractable epilepsy (see [84,85]). Additionally, VNS and other neurostimulations like deep brain stimulation (DBS) and responsive neurostimulation (RNS) have also been used to treat DRE in off-label applications [86]. Please see [86] for a systematic review and meta-analysis reviewing and comparing the efficacy of VNS, RNS, and DBS in generalized epilepsy.

VNS involves the implantation of a programmable pacemaker-type device (pulse generator) in the subcutaneous chest wall, which sends continuous signals with on-demand capabilities to the left (preferably) sensory afferent fibers of the Vagus nerve [87,88,89]. However, see [90] for a discussion comparing left and right VNS. This therapy is theorized to affect multiple brainstem nuclei due to its direct and indirect synaptic connections to areas like the Nucleus Tractus Solitarius (NTS), Locus Coeruleus (LC), reticular formation, and raphe nucleus, which advance their synaptic activity and neuromodulation via myelinated A and B fibers to cortical regions involved in epileptogenesis [89], like the hippocampus and posterior cingulate [87,88]. While considered relatively conservative, side effects and risks exist. Most notably, the device is rarely removed; others include post-surgical infections, vocal cord paresis, facial involvement (weakness), cough, potential nerve damage with continuous stimulation [83], and cardiac changes [91]. As a result of the potential risks and availability of invasive VNS, several non-invasive (transcutaneous VNS—tVNS) devices are available. See [88,91] for a review on tVNS. A systematic review and meta-analysis on the pediatric application of tVNS concluded that while it does not result in complete seizure remission, a 50% responder rate is possible and more likely in patients with fewer attempted ASMs and a later age of seizure onset [92]. Treatment length for invasive and non-invasive VNS ranges from 12 weeks to 2 years [91].

Trigeminal nerve stimulation (TNS) is an alternative non-invasive treatment application for epilepsy. Trigeminal nerve stimulation (TNS) for the reduction of seizures in PWE and especially persons with DRE has become an attractive alternative to other types of stimulation, such as VNS and deep brain stimulation (DBS), which are somewhat irreversible, cost prohibitive, and have an estimated efficacy of 50% [61]. While invasive, responsive neurostimulation (RNS) is a reversible, closed-loop neurostimulation designed to continuously monitor and react to the individual’s neural activity [93]. The conceptual model for this unit was derived from defibrillator machines used for converting sudden cardiac arrhythmias [93]. Please review [93,94,95] for more information on RNS. Additionally, please review [96] for comparisons between VNS, DBS, and RNS.

TNS is a non-invasive transcutaneous neurostimulation transduced from a small mobile device using small electrical adhesive pads placed externally and bilaterally on the participant’s forehead or under the eye area over the maxillary bone (supraorbital and infraorbital foramen). Interest has been growing in this technique since a pentylenetetrazol (PTZ)-induced seizure animal model was subsequently rescued with the acute application of TNS [84]. Following that study, the authors reported on the first two humans to be treated with TNS [97], and later, they included seven participants in a proof of concept trial in the treatment of DRE with TNS [98]. The application and targeting of the trigeminal nerve (TN) for treating epilepsy is neurobiologically sound. The TN projects extensively to the NTS and the Locus Coeruleus (LC) [98], which broadens their impact by disseminating projections centrally [99]. Of particular interest is the LC, located just anterior to the trigeminal accessory nucleus sitting in the fourth ventricle; it sends robust projections to the ventral and dorsal regions of the hippocampus, thus directly connecting to and influencing temporal lobe activity [61]. As described below, the TN sends projections to large areas of the pons, reticular formation [61], midbrain, spinal cord, and thalamus.

Individuals considered for neurostimulation trials have been unsuccessful in their seizure control with two ASMs over one year [100]. Efficacy is variable, ranging from 30–50%, depending on the sample size and the study. We suggest reviewing work by Gil-López and colleagues [61] for a summary of the favorable response rates in the available studies. Side effects of the treatment consist of headaches, skin irritation, and mild to moderate anxiety [100]. Noncompliance is problematic due to the time required, 8–12 h per day for months, to achieve therapeutic results [61].

Our list of treatments is by no means exhaustive or complete. However, we have provided what appear to be contemporary therapeutic approaches and have supported efficacy in the literature. As epilepsy and seizures, described previously, can be an all-consuming reality affecting not only the sufferer but also the entire family matrix, other, likewise complementary, interventions for seizure control exist, such as photobiomodulation, nicotine patches, hyperbaric oxygen therapy, and repetitive transcranial magnetic stimulation (rTMS).

## 5. Cannabis and Epilepsy

The use of the Cannabis plant was first recognized in 12,000 B.C.E. in central Asia, for making rope, seed production, and its high fiber content [101], and in India, for the production of resin and its many uses [102]. Later signs of cloth made of hemp exist [101]. In contrast, in other regions like Africa and the Middle East, Cannabis was recognized for its psychoactive properties [102]. Yet, it was the Chinese emperor Shen Nung in 2700 B.C., considered by many as the father of Chinese medicine [101], who was acknowledged for uncovering Cannabis’ first medical therapeutic applications [102]. Remarkably, the Sumerians around 1800 B.C.E. reported treating “night convulsions” with this versatile plant [102]. More recently, in the 1850s B.C.E, it was documented in both Greek and Arabic medical texts in the treatment of seizures and epilepsy [103]. In 1843, a chemistry professor and physician, W.B. O’Shaughnessy, documented the effects of Cannabis Indica on many animals and, notably, for treating a young baby’s seizures [104]. From here, Cannabis was placed in future physicians’ consciousness for the treatment and modulation of epileptic seizures resistant to drug therapy, which at the time was the class of Bromides [102,105].

The human brain contains a potent neurotransmitter modulator known as the endocannabinoid system (ECS) [42]. The ECS consists of three chief molecular components: receptors (CB1 and CB2), ligands (AEA and 2-AG), and enzymes [106]. This system is highly specific in its signaling and neuronal-type expression and has a high prevailing spatiotemporal specificity for synthesis and breakdown [42]. Endocannabinoids (eCBs) are classified as lipid-derived messengers that play a crucial role in inhibiting presynaptic neurotransmitter release (retrograde modulation), thus playing a formative role in both physiological and pathological states [42]. However, this system becomes dysfunctional and modified in PWE and other more benign aberrant physiological states, such as stress [107]. An example of this dysregulation is when the CB1 receptor, the most abundant presynaptic cannabinoid receptor in areas of the brain such as the hippocampus, basal ganglia, and cerebellum, becomes downregulated during the acute seizure stage, yet rapidly shifts to an upregulated state in the chronic phase [107]. It is accepted that epilepsy is an expression of a rhythm network problem. Still, other rhythm network aberrations exist in the brain, like tinnitus, autism, AD, PD, depression, schizophrenia, and essential tremor [107]. The brain’s neuronal networks are never silent and therefore display various synchronized oscillatory frequencies and patterns that control memory consolidation (sharp waves), resting states, and physically active states [107]. Due to eCBs’ wide range of effects on the CNS, PNS, and immune system, the system also contributes to learning, energy metabolism, pain, reward, and emotional state [108].

The use of Cannabis in the treatment of epilepsy, as stated above, has been documented for thousands of years. Yet, it was not until 2018, after multiple clinical trials initiated in 2013, that the FDA approved the first-ever concentrated and purified form of Cannabidiol (CBD) for Lennox–Gastaut syndrome (LGS) and Dravet syndrome (DS), two rare forms of epilepsy [103]. In a 2014 interventional study, the efficacy analysis revealed a reduction of 35.6% in motor seizures over the 12-week trial period; this effect was higher in DS, at nearly 50% [103]. Additionally, caregivers were more likely to observe and report general improvement compared to placebo [103]. Later, in 2017, a moderate-sized, multicenter, placebo-controlled trial was conducted, concluding that 43% of the enrollees in the CBD (as an add-on to existing antiseizure medications) group experienced at least a 50% reduction in seizure frequency (95% CI, 2.00 odds ratio [109]. The interventional group also exhibited increased adverse reactions and participant withdrawals compared to placebo [109]. Another large 14-week trial of 225 patients diagnosed with LGS showed a 36–40% reduction in drop seizures with 10 and 20 mg of CBD, respectively (*p =* 0.0030 and *p =* 0.0006) [109,110]. Common adverse reactions are diarrhea, drowsiness, loss of appetite, elevated levels of liver enzymes (in patients on ASMs) [103], convulsions, and vomiting [102]. Taken together, these studies, for the first time, showed class 1 evidence for the adjunctive use of CBD in two severe forms of epilepsy [102,109]. For more on CBDs efficacy, see [103,111,112,113].

Cannabinoid signaling is complex and is beyond the scope of this paper. However, a few takeaways concerning epilepsy shall be noted. As eCBs are involved with controlling neurotransmitter release and modulating synaptic transmission, they have proven to be robust, immediate, and “on-demand” messengers that can promote or terminate neuronal hyperexcitability [113]. Several mechanisms are proposed: CBD is understood to act on GABA_A_ receptors by promoting their known rapid inhibitory effect on downstream neural activity, by inhibiting NMDA receptors, and modulating the psychogenic properties of THC while enhancing THC’s anticonvulsant properties (entourage effect). Several mechanisms are proposed: CBD is understood to act on GABA_A_ receptors by promoting their known rapid inhibitory effect on downstream neural activity, by inhibiting NMDA receptors, and modulating the psychogenic properties of THC while enhancing THC’s anticonvulsant properties (entourage effect) [106]. Also of interest is the participation of the TRP (transient receptor potential) cation channels. CBD acts as an agonist to TRPV1 and TRVPA1 while acting as a TRPM8 antagonist. These ion channels are the same as those found in the respiratory and olfactory epithelium, and their relevance will be discussed later.

In summary, even though an ongoing knowledge base of CBD’s physiological mechanisms has materialized, complete, intimate knowledge of the spatiotemporal dynamics of eCBs is still lacking, and the current therapeutic application of exo-cannabinoids for epilepsy is realistically in its infancy. To illustrate briefly, eCBs have been shown to inhibit seizure activity at lower concentrations, while higher concentrations promote it [113]. Based on more current reports [42,108,113], considerable insight is nevertheless needed for precise, predictable, safe, and effective treatment administration. Additionally, it is uncertain whether the effect of CBD pharmacokinetics alone disrupts seizure signaling or whether it is from complex metabolic interactions with the prescribed ASMs, such as clobazam [109]. With that said, CBD is a hopeful and necessary adjunct to the treatment of seizure disorders. Please review [42,103,106,112,113,114,115] for discussions underlying CBDs’ physiological and pharmacodynamic properties, relevant understanding of CBD, and its role in epilepsy.

## 6. CO_2_ and Chemosensitivity

The anticonvulsant therapeutic effect of CO_2_ was first recognized in 1928 when Carbogen containing 10% CO_2_ was administered to people with petit mal epilepsy [116]. Since then, animal and human trials using varying concentrations of Carbogen have demonstrated the positive anticonvulsant effects of CO_2_ as quickly as 30 s after administration [116,117]. Lennox, in 1936, showed reduced EEG spike-wave activity using 10% CO_2_ [118], while Pollock, in 1949, applied a range of 15–30% carbon dioxide in which all concentrations of CO_2_ prevented electrically induced seizures [119]. Woodbury et al., in a mouse model, showed protection from electrical and drug-induced seizures with carbon dioxide administration [120]. Meyer et al. also found an inhibitory effect on seizures in monkeys and cats with carbon dioxide inhalation and theorized the therapeutic combined mechanism of action of CO_2_ was (1) to cause a shift in intraneuronal membrane potentials and (2) to allow for increased accessible blood O_2_ via its inherent ability to dissociate oxygen from hemoglobin [121]. Further, Ohmori et al. demonstrated 10% carbon dioxide inhalation in a *Scn1a* missense mutation hyperthermia-induced seizure-susceptible rat model resulted in strong seizure suppression within 10 s, while Schuchmann et al. [122] observed seizure suppression with 5% CO_2_ [123]. Interestingly, the _p_CO_2_ measured had not reached physiological levels when seizure suppression occurred, indicating that a total homeostatic return of _p_CO_2_ is not required [123]. In a small human pilot study by Yang et al., twelve individuals with a diagnosis of childhood and juvenile absence seizures were treated with room air, 5% CO_2_, and 100% O_2_ in a hyperventilation-induced absence seizure model. The results showed a reduction in spike-and-wave discharges and reduced seizure occurrence in the Carbogen group alone [124].

Based on these studies, others have continued their investigations into understanding CO_2_’s potential mechanism of action, such as elucidating CO_2_’s role in reducing overall cortical pH and increasing cortical acidosis within seconds of administration. One conceivable mechanism for this was discovering and understanding the acid-sensing ion channel-1a (ASIC1a). First cloned in 1997, three of the four variants are found to be highly sensitive to pH changes both intra- and extracellularly [125]. See [125,126] for more on the molecular structure of these ion channels. ASIC1a ion channels constitute a considerable proton receptor in the human brain, such as in the hippocampus [127], amygdala, somatosensory cortex, cingulate cortex, and the striatum [59]. Of all the ion channels, it is the most sensitive to changes in pH, and its existence modulates neuronal excitability, seizure progression, and termination [59,128]. This ion channel is highly conserved from mouse to human, with a 3-fold increase in cellular surface trafficking in humans, making it an integral player in preventing acidotic neuronal injuries [127]. The existence of this ion channel is reliably shown in animal studies to enhance seizure termination, and the absence of it is associated with prolonged and increased seizure severity [127]. It is believed that in an acidic environment, this ion channel upregulates overall neuronal inhibitory tone and generates notable ASIC1a currents, as Zeimann et al. showed in mice hippocampal slices, revealing its meaningful role in seizure termination [128].

Another crucial ion channel is the NBCe1 (*Slc4a4*), a sodium bicarbonate transporter that is robustly present in the mammalian brain, such as in the piriform cortex, cerebellum, hippocampus, and olfactory bulb [63]. Numerous animal and human studies have linked deficiencies in NBCs to the pathophysiology of seizure induction, progression, developmental disorders, and migraine [63]. Mouse models show us that severe metabolic acidosis occurs without NBCe1 (*Slc4a4*) and is incompatible with life [129].

The unique and potential effectiveness of CO_2_ on neuronal hyperexcitability is interconnected to the system’s pH, whether it is blood pH or neuronal pH. Changes in pH occur before, during, and after seizure activity [55]. Initially, the extracellular pH becomes more alkaline, theorized to be the result of increased local bicarbonate (HCO_3_^−^) levels, which themselves result from increased GABA_A_ receptor activation, both of which urge the neurons into a state of hyperexcitability [55]. Yet, as the seizure progresses and the intraneuronal pH becomes more acidic, an extracellular biphasic shift closely links with acidosis; this is presumably the result of CO_2_, lactic acid, and H+ [130] actively transported out of the progressing intraneuronal acidic environment due to increased neuronal metabolism [130,131]. This intraneuronal acidosis is thought to initiate seizure termination via a cascade of anticonvulsant effects [55]. However, in refractory seizures, the extracellular acidosis is brief, partly due to local astrocyte activity, and the system remains in a state of alkalosis and, therefore, persistent hyperexcitability. This is problematic as neurotransmitter receptors, ion channels, biochemical byproducts, and cascades of synaptic transmission are all highly sensitive to pH homeostasis, which is fundamental for optimal brain function [130]. Brainstem chemoreceptors are acutely sensitive to CO_2_ and pH changes and respond by altering the respiratory network’s activity to rectify and manage rapidly fluctuating cortical pH and maintain neuronal fidelity [63,130]. This is accomplished by brainstem astrocytes, albeit physiologically acting in reverse of cortical astrocytes; they utilize the NBCe1 channel to alter the surrounding pH by activating nearby respiratory networks via ATP release and then modulating breathing rate accordingly [130]. In fact, without CO_2_, respiratory rhythm-generating circuits are silent and necessitate a specific level (threshold) of CO_2_ to function [129,132].

The formation of the respiratory central pattern generator enables autonomous breathing, which comprises three distinct stages: inspiration, post-inspiration, and expiration [133]. A “triple oscillator hypothesis” has been proposed that encompasses the neuroanatomical and neurochemical substrates responsible for the baseline microcircuitry that perpetuates the breathing network [133]. Specifically, the preBötzinger Complex (preBötC), a discrete area located in the ventral lateral medulla that initiates inspiration, the Bötzinger Complex (BötC), the post-inspiratory complex (PiCo), and the expiratory oscillatory region in the rostral medulla (retrotrapezoid nucleus and parafacial respiratory cluster) [133] are postulated to act as the rhythmic respiratory network in eucapnia and hypercapnia states [134,135]. These neurons utilize a variety of glutaminergic, glycinergic, and cholinergic inputs, express several neurochemicals like somatostatin and neurokinin-1 receptor (NK1R), and contain chemoreceptors like adrenergic C1 cells to generate and maintain autonomous rhythmic breathing [133]. However, this model is absent of pontine aspects and is suggested by Dhingra et al. to be an inadequate explanation for eupneic breathing. Further, they suggest erroneous interpretations of previous studies have occurred because they are based on “anatomic and electrophysiological single neuron resolution and not on population scale activity” [136]. Please see [137] for more on this current debate.

Nonetheless, the intermingling of central nervous system structures and networks, as well as the brainstem astroglial system, is theorized to create a “distributed central chemosensitivity” functionality that is sensitive to CO_2_ and, therefore, participates in regulating the respiratory response [132]. While current central respiratory models place the retrotrapezoid nucleus (RTN), which, in lesioned models, substantially diminishes the respiratory response to CO_2_ [129], and the medullary raphe at the center of the pH-sensing system, a more likely hypothesis is this “distributed network”, which encompasses the sites as mentioned above, contributes to and integrates CO_2_ inputs and, thus, respiration control and pH maintenance, including both blood and brain parenchymal p_CO2_/pH [135]. Several studies are congruent with this understanding. For example, research suggests that the preBötC contributes 20–25% of the ventilatory response to CO_2_, and the associated glial cells contribute 20–25% to central CO_2_ chemoreception, with the latter seemingly crucial for breathing when there are physiological stresses such as hypercapnia or hypoxia [132,135,138]. Moreover, the preBötC located in the ventral medulla is said to be readily responsive to a continuum of alternating ratios of neuronal states—silent, tonic, and bursting—resulting in an inspiratory rhythm that is uniquely “stable in its rhythmogenesis” [135]. Unlike other networks, the preBötC, because of its heterogeneous cellular properties, responds dynamically to changes in neuromodulation and synaptic excitability [135]. The preBötC ability for synchrony under ever-changing conditions, along with its mono- and-oligo-excitatory and inhibitory (mostly running in parallel) synaptic connections to the NTS, contralateral preBötC, BötC, NTS, RTN, parafacial region, parabrachial, Kölliker-Füse nuclei, parahypoglossal, and periaqueductal gray, and to some extent the limbic, hippocampus and prefrontal areas, modulates breathing in tandem with and as a result of emotional, cognitive, and basic physiological behaviors via its exceptional “binding activity” across the brain and brainstem [139,140].

Respiratory CO_2_ appears to have a more robust triggering response than metabolic CO_2_ [129]; while previously thought to play a secondary (by proxy) role in chemosensitivity to H^+^ ions, presently, studies suggest CO_2_ and its components H^+^/HCO^−^_3_ independently act, and with greater importance, on the central chemoreflex [141]. Conceivably, this relates to an evolutionary challenge. When mammals transitioned from sea to land, preserving a stable pH became problematic. With 7× more O_2_ in the air than in water, a lower ventilatory response was necessitated, which resulted in a higher blood concentration of CO_2_ [129]. Metabolically, the human brain creates about 75 L of CO_2_ daily, roughly 20% of the total corporeal production [129]. As previously stated, maintaining the pH homeostasis and avoiding irregular fluctuations is crucial for stable neuronal activity, and the inability to do so is associated with numerous brain and neurological disorders like epilepsy, schizophrenia, and Alzheimer’s disease [129,142,143]. Please review [129,141].

Historically, breathing in humans has been broadly recognized to be largely controlled by central CO_2_ chemoreception, and, therefore, primarily within the domain of the brainstem [144]. Yet, the cortex’s role in breathing and its direct effects on the cortex are gaining interest [145]. Stimulation studies of the amygdala, hippocampus, and insula, along with neuroimaging studies, EEG, and transcranial magnetic stimulation (TMS), have revealed areas in the motor strip, including the premotor and supplementary motor regions, to have a direct effect on breathing [145]. Furthermore, intracranial EEG (iEEG) monitoring during surgery for partial epilepsy reveals a correlation between limbic and cortical activity and the breathing cycle, with nasal breathing showing greater iEEG cortical coherence [145]. Nasal respiration is hard-linked to respiratory drive and cortical activity [146]. The olfactory epithelium propagates local field potentials (LFPs) to the olfactory bulb (OB) in a respiratory-phase locked propagation [146]. The airflow rate, not the respiratory rate, can alter the OB responses via the changes in the sensitivity of OB glomeruli [146]. The air moving through the nose has a mechanical effect on the olfactory sensory neurons; thus, this potential “integrator” relies on an intact OB to produce this natural respiratory rhythm that, through direct efferent projections, projects to the piriform (olfactory) and entorhinal cortices; the latter then projects to the hippocampal region [146,147,148]. Nasal inspiration is coupled with peak cortical oscillations; this activity is reduced when breathing switches to mouth breathing [148]. As continued maintenance of the optimal “physiological acid–base balance” is necessary for normal cellular function, respiration can serve as an activity integrator or a “master clock” across all brain regions [147] as the respiratory rhythm is thought to synchronize cortical networks and temper cortical excitability [148]. The result is the entrainment of the cortical neural networks that specifically encode “olfactory coding, memory, and behavior” [148].

A detailed review of the pathophysiological aspects of seizures is beyond the scope of this paper. Please see [55] for a thorough review of the potential pathophysiological mechanisms and physiological metabolism involved in seizure perpetuation and [149] on ion dynamics during seizures. Additionally, please review [131] for an understanding of astrocytes’ promising role in maintaining extracellular homeostasis pH via bicarbonate shuttling. See [150] for a detailed review of the therapeutic importance of acid–base balance based on a physiological perspective.

The word semiology (the English adaptation), uniquely utilized in the context of epilepsy [151], originates from the Greek word “semeion,” meaning a sign [152], to make known, or the observed signs “contained in an object” [153] or, in this case, a disease like epilepsy. Epilepsy is not a focal brain lesion but originates from many known areas, like the frontal lobes, temporal lobes, cingulate [58], piriform cortex [154], and hippocampus [13]. Now, subcortical structures like the basal ganglia and thalamus are identified as epileptic regions [151]. Historically, recognizing different types of seizures based on their outwardly expressed and distinct phenomenology has been central in defining type and diagnosis. In other words, the clinical manifestations are the result of specific, non-haphazard neuronal activity within each interconnected substrate and its ensuing cadenced cohesiveness, which ultimately shows the characteristic physical signs involving motor, somatosensory, levels of consciousness, behavioral, and autonomic patterns [151]. The idea that epilepsy should encompass a neural network focus versus simply an “epileptic focus” becomes evident. See [151] for an overview of seizure semiology.

As described previously, SUDEP is a potentially devasting outcome of seizures and is reported to represent 10–50% of all deaths in people with intractable epilepsy [51]. Based on the information provided in this paper, we submit that the respiratory mechanism involved in SUDEP perhaps illustrates a partial model or link between the complex anatomical substrates involved in seizures and the intricately involved central and peripheral nervous pathways (systems) that are integral for allowing our interaction with the external environment for our survivability as land-bound, air-breathing mammals. To illustrate, the brainstem contains neurons that are sensitive to CO_2_, chemoreceptors, like the multitude of serotoninergic neurons found throughout the mesencephalon and medulla, which respond to elevated CO_2_ levels by increasing respiration, specifically by binding to 5HT_1A_ receptors [41]. Not only is the brainstem responsible for maintaining adequate respiration, as mentioned, but cortical structures like the hippocampus, amygdala, and orbitofrontal region, which are sites commonly involved in seizure activity, also contribute rostrocaudal projections to lower brainstem breathing circuits [38]. This is not far-fetched, as our breathing needs to be coordinated and controlled during conscious activities such as laughing, singing, and speaking [38].

Both focal and GCTSs have been shown to have depressed hypercapnic ventilatory responses (HCVRs), indicating decreased chemosensitivity [155]. Further noted is prolonged hypoventilation in persons with generalized convulsive seizures (CGSs) [155]. Sainju et al. found an inverse relationship between the HCVR slope and CO_2_ [156]. This means that the larger the slope, the greater the response to low levels of end-tidal CO_2_ with amplified inherent ventilatory reactions, which may be largely genetically influenced [156]. They found that individuals with weakened CO_2_ central respiratory drives had an increased risk of persistent hypoventilation and, thus, speculated SUDEP rates and “interictal respiratory variability predicts the severity of postictal hypoxemia” [157]. We urge the reader to review [37,38,41,51,155,156].

## 7. Nasal Anatomy and Pathophysiology

This seemingly innocuous protruding appendage plays a vital role in human immune function, olfaction (smell), respiration, immunological threat detection, voice characteristics [158], air manipulation, and, realistically, overall survival [159]. Interestingly, even the classical embryological understanding of this structure does not fully explain its importance [160]. In fish, the nose is solely an olfactory organ, yet in tetrapods, including their ancestors and descendants, the advancement and addition of the respiratory nose creates an “intricate and elaborate” complex (dual) organ that is not only entwined and linked remarkably from quadrupedal to bipedal gait but also human behavior and development [160]. For an interesting read on the evo-devo embryological nose theory, see [160,161].

The anatomical structures of the face and nose arise from three embryonic tissues: the ectoderm, mesoderm, and neural crest, and at four weeks of gestation, the structures that develop into the future face are perceptible [162]. The human nasal cavity comprises two air spaces created by three bony turbinates (superior, middle, and inferior), which begin to develop in the sixth week [163]. Also referred to as concha, these anatomical structures, formed by the 16th week of gestation and ossified by the 24th week, are located at the rear of the nasal cavity and are profuse with glands and a robust blood supply [159,162]. At the 24th week of gestation, the nose’s development is almost complete [162]. The olfactory epithelium, developing from the ectoderm, starts to differentiate into the olfactory receptors and eventually into the olfactory bulb in the frontal lobe [163]. Notably, the primary olfactory centers in the brain develop simultaneously [160]. As such, the rhinencephalon—the primary olfactory region [164]—develops relatively early in brain development; this region includes the olfactory bulb and tubercle, gyrus subcallosus, septum lucidum, hippocampal formation, piriform cortex, and rostral perforated substance [164]. Finally, the chemoreceptive capabilities of the nose are established during the third trimester. However, postnatally, the nasal cavity still undergoes changes and growth [70]. To illustrate, from ages three to five, the turbinate’s size (and thus changes in airflow dynamics) is estimated to double with continued morphological maturity well into the mid-teens [70]. Furthermore, respiratory diseases can arise during this time, altering nasal physiology and functionality, which becomes pertinent when administering any type of intranasal intervention [70].

At first glance, the nose may appear to be a characterless empty vault with the mundane job of transporting the advancing air. However, it is a three-dimensional complex and fascinating structure that is robustly represented and explored in the literature concerning human health, disease, and pharmacological manipulation. The fundamental layout of the nose consists of two irregularly shaped cavities formed by the presence of a septum (anteriorly cartilaginous and posteriorly boney) [165], a roof formed by the ethmoidal cribriform plate, and two posteriorly positioned lateral walls containing the three turbinates [70]. Air and its constituents enter the anterior portion of the nose, known as the vestibule, lined with squamous mucosal epithelium and nasal hairs, and passes through the external nasal valve, which regulates the velocity and direction of airflow into the main chamber of the nose [70,165]. Here, respiratory mucosa covers the largest area of the cavity, while the olfactory mucosa inhabits the posterior superior portion of the cavity [70]. The posterior section contains the nasal–pharynx lymphoid tissue (NALT), which links the nasal cavity to the nasopharynx [70]. For a detailed account of nasal embryology see, [163].

While the essential anatomical players and topography of the nose are similar across species, specific differences need to be recognized regarding airflow, immunity, olfaction, and respiratory function [166]. Humans are considered microsmats, which rely less on the olfaction function and more heavily on their respiratory abilities. In contrast, rats and dogs fall into the macrosmatic category, depending on their enhanced primary olfactory capacity for survival [166]. Notably, the nasal compartments in humans, dogs, and monkeys are entirely segregated into two chambers with no communication. In contrast, a small septal window in the rat establishes an intermingling of the two cavities [166]. This dissimilarity is relevant when applying comparative research studies and considering the importance of cortical integration and activity in the central nervous system. A detailed narrative of the species-specific differences is beyond the scope of this paper, and we refer the reader to [166]. However, we will highlight some essential differences below.

One of the fundamental distinctions is the turbinates. For example, the number, placement, and complexity will vary across species. In humans, the three paired turbinates have several functions. First, their bony shelf-like structure creates the three passageways below, called the meatuses: superior, middle, and inferior. These allow for inspired air to travel (nasal airflow), are responsible for the airflow pattern and direction, and, by their positioning, affect toxicant and particle accumulation [166]. The inferior meatus is the largest in humans. It is where the majority of inspired air flows, traveling along the ventral two-thirds of the nasal floor to the posterior nasopharynx [167]. These paired turbinal outpouches of the nasal, ethmoidal, and maxillary bones increase the surface area to support circulation, mucus secretion, odorant deposition and processing [168]. Additionally, nasolacrimal and palatine ducts, accessory olfactory organs, and four paired paranasal sinuses add supplementary functionality to this system and are somewhat species-specific [166]. Again, please see [166] for details.

Four distinctive types of epithelial exist throughout the nasal cavity in different proportions: squamous, transitional, respiratory (RE), and olfactory epithelial (OE) in humans, dogs, and research animals. Their distinctions highlight their physiological functions. Most significantly, the pseudostratified ciliated columnar mucosa membrane’s respiratory epithelium envelops 75% of the nasal area [166]. It is populated substantially with a superficial blood supply, basal cells, goblet cells, and ciliated and non-ciliated columnar cells that support local immune capabilities, cleaning, temperature management, and humidity control [168]. The second most abundant epithelial type is the olfactory epithelium, occupying close to 50% of the nasal surface area in the rat but, in man, accounting only for 3% while being constrained to the superior posterior cavity and septum in the latter [166]. The junction between these two epithelial tissues (RE and OE) is less defined in humans and animals, and its randomness increases with age. Furthermore, with injury in this region, the OE, being more susceptible to injury because of the lack of ciliated epithelium, will become increasingly populated with the less discriminating RE [166]. It is clear that intranasal topography is highly specialized and critical for survival; this is further supported by the fact that this region is significantly conserved across all species and characterized by the olfactory sensory neurons (OSNs), supporting cells and basal cells in all mammals [166].

The basic configuration is a basement membrane in which the OE lies; this is supported by the lamina propria, which is embedded robustly with a vascular and lymphatic supply, immune cells, nerves, and glands [166]. The basal cells can be seen as a type of stem cell that perpetuates OSN regeneration, and the OSNs’ bipolar neurons have unique properties that allow for the detection of specific odorants, which then travel to specific cortical and brainstem regions [166]. The OSNs’ axons form the olfactory nerves within the OE, which pass through the superiorly lying ethmoidal cribriform plate to the olfactory bulb (OB) [70]. The tufted cells within the OB send efferent signals to the olfactory cortex [70]. It is here, at the OSNs, that the outside world, whether air, CO_2_, toxins, viruses, or drugs, is in direct contact with the CNS, notably bypassing the thalamus for integration, consequently having a direct and somewhat immediate impact on the overall neurological state of the brain [70].

In addition to the OSNs, in the anterior septal and anterior lateral nasal walls, two branches from the trigeminal nerve (TGN) innervate the RE and the OE [70]. This nasal trigeminal nerve system contains a group of specialized receptors that distinguish them by their capacity to process chemosensory information separate from olfaction [169]. These nasal trigeminal fibers are unique as they lack a squamous epithelial covering and respond acutely and directly without injury to direct chemical stimulation [169].

The sphenopalatine artery and ethmoidal branches from the ophthalmic artery (a branch of the internal carotid artery) [170] enter through the cribriform plate and are congruent with a matching venous supply, infiltrating the nose and providing circulation [70]. Small facial arterial branches supply lesser nasal regions, and the venous return is through the facial vein anteriorly and posteriorly by the sphenopalatine veins into the pterygoid plexus, while the submandibular nodes manage the lymphatic drainage [170]. The incoming blood flow moves against (anteriorly) the slow-moving mucus secretions, generally moving caudally and posteriorly toward the nasopharynx [70,166]. Please see [70] for more clarification and [166] for comparative histological differences and anatomy details.

The nasal parasympathetic innervation originates from the superior salivatory nucleus in the pontine tegmentum’s tractus solitarius [171]. From here, and ensheathed separately within the brainstem’s pontomedullary reticular formation, the afferent and efferent fibers carrying visceral motor, general, and special sensory information pass lateral to the branchial motor fibers of cranial nerve seven, identified as the nervus intermedius [172,173]. As the facial nerve travels along the petrous portion of the temporal bone, the geniculate ganglion arises, containing the cell bodies for special sensory (taste) and somatic sensory regions of the ear [173]. It is here at the geniculate ganglion where the chorda tympani and greater petrosal nerve deviate on their paths: the former will travel up the foramen lacerum to join a portion of the TGN, V2, and the deep petrosal nerve [174].

The Vidian nerve (nerve of the pterygoid canal) consists of the greater petrosal (pre-ganglionic parasympathetic) and deep petrosal (post-ganglionic sympathetic) nerves before entering the pterygoid canal and exiting to reach the pterygoid palatine ganglion (PPG) or the sphenopalatine ganglion (SPG) [174,175]. Within this bundle, the sympathetic fibers of the deep petrosal nerve, initially arising from the upper thoracic spinal cord’s intermediate gray horn via the internal carotid plexus, join and travel along the sensory fibers of the maxillary division (V2) of the TGN [174,175]. The sympathetic fibers do not synapse in the PPG as they have already synapsed in the superior cervical ganglion. Still, the parasympathetic fibers synapse here before innervating the nasopalatine mucosa to control vasomotor and glandular activities [175]. Interestingly and anatomically, cranial nerve seven (facial nerve) and its subdivisions utilize the trigeminal nerve as the thruway to subserve autonomic functions of the nose [171]. Other autonomic concerns like eye irritation and gustatory responses affect the superior salivatory nucleus, and the hypothalamus strongly influences autonomic control here. Olfactory information converges in the hypothalamus and other physiological functions linked to executing behavioral responses [176].

Variations in nasal passage airflow (congestion and decongestion) occur throughout the day with an individualistic but regular alternating cyclical phase of dominance lasting from thirty minutes to six hours within a 24 h cycle and, thus, considered an ultradian rhythm [177]. Known as the nasal cycle, it is the result of quantifiable alteration and lateralization of autonomic nervous system activity upon the nasal venous plexus residing in the nasal submucosa [178] of the anterior septum and inferior turbinate [179] and within the ethmoidal sinuses [177]. This nasal rhythm has long been theorized to dictate and express hemispheric cortical laterality, specific human behaviors, and brain function [180] and is shown to be altered depending on body positioning, age, estrogen levels [177], and possibly handedness [179,181]. Specifically, the reciprocal alternating nasal cycle declines with age and has a longer periodicity in sleep [182], and nasal patency (decongestion) is greater in the nares contralateral to the side of the lateral recumbent position [182]. Alterations in the nasal cycle have been associated with many neurological diseases, like autism, Parkinson’s, schizophrenia, cord injuries, and cardiac dysfunction [179]. See [179] for a list of references. The physiological mechanisms, cortical implications, and role in human health and disease behind this mammalian reflex are still being investigated. Research has supported the mechanistic idea of enhanced air conditioning and humidification, increased mucociliary clearance, and protection against unwanted infection [179]. Yet, there is support for enhanced olfaction function aimed at predator defense and food optimization, which can occur through an optimal nasal passage odorant solubility, depending on the odorant’s solubility preference and the nostril’s patency status at that time [179].

Additionally, the activation of specific cortical regions has been identified and recorded for specific olfactory and trigeminal stimulants. Kollndorfer et al. utilized fMRI to evaluate the central processing of known trigeminal agonists, CO_2_ and cinnamaldehyde, and an olfactory agonist, menthol [183]. They concluded three networks—the olfactory network, the somatosensory network, and the integrative network—were activated by both agonist types, yet, temporally and chemically, slightly favored one over the other, theorized to be based on functionality and behavioral responses associated with the stimuli. For example, the olfactory network processed all three stimuli presented unilaterally in the left nostril, primarily in both hemispheres, and with a temporal delay of the trigeminal agonist CO_2_; the somatosensory network significantly favored the right hemisphere. In contrast, the integrative network revealed left hemispheric preponderance [183]. These findings of overlapping but distinct central processing of intranasal olfactory and trigeminal stimuli align with a meta-analysis using an activation likelihood estimation tool by Albrecht et al. [184]. Using CO_2_, thought to be a unique trigeminal stimulus (although some reports suggest integrated activity with the olfactory network in the piriform cortex), mapping of the chemosensory networks showed commonality in the right piriform cortex, orbitofrontal portions, insula, and middle frontal gyrus [184].

## 8. Trigeminal Nerve Anatomy and Correlates

The trigeminal nerve (TGN), derived from the first branchial arch and designated the fifth cranial nerve, is the largest [185] and longest [70] of the face and connects to the brainstem in the mid-lateral pons [173]. As its name implies (the three twins), it is comprised of three divisions: the ophthalmic nerve (V1), the maxillary nerve (V2), and the mandibular nerve (V3) [185]. In the floor of the mid-cranial fossa, a depression referred to as Meckel’s cave houses the large semilunar sensory ganglion (Gasserian ganglion) in which the three branches emerge [173]. This large trigeminal sensory ganglion (TG) houses numerous (20,000–150,000) pseudo-unipolar cells that have two axonal branches exiting centrally and distally from their soma, suggesting both orthodromic (afferent) and antidromic (efferent) conduction activities [186,187]. From here, it is the trigeminal root that extends and connects the trigeminal ganglion to the pons; this region is recognized as a transitional territory because central myelin replaces the peripheral myelin and is susceptible to compressive injuries [186]. At the pontine brainstem, central processing of the TGN afferents begins and is known as the trigeminal sensory complex or, more fittingly, the trigeminocervical complex (TCC). The TCC houses the second-order neurons and extends from the caudal mesencephalon to the upper regions of the spinal cord from C2–C4 [186]; here, it becomes continuous with the dorsal root ganglia of the spinal cord [173,187]. Just superior to the TCC is the mesencephalic nucleus, denoted from the level of the superior colliculus in the rostral mesencephalon to the mid-pons, thereby creating three distinguishable nuclei throughout each brainstem region, notwithstanding their indistinct borders: the mesencephalic nucleus, the pontine nucleus, and the spinal nucleus [173]. In addition, a smaller motor root sits just distally to the sensory root in the pons and supplies the muscles of mastication [173].

All three divisions of the trigeminal nerve—V1, V2, and V3, and their 3–14 individual branches—carry sensory information for touch, pressure, thermal [187], vibration, proprioception, and nociception and adhere to a topographic and a somatotopic distribution throughout, including their tracts and nuclei [186]. In general, proprioceptive fibers pass to the mesencephalic nucleus, discriminative touch (low-threshold mechanoreceptors) to the pontine nucleus, and finally, thermoreceptor and nociceptor information are transmitted down to the spinal trigeminal nucleus [186]. The latter nucleus is further subdivided as it descends the brainstem into the pars oralis, interpolaris, and the subnucleus caudalis, rostral to caudal, respectively, while also sending off collaterals to areas in the reticular formation that will coordinate with visceral and autonomic responses [173,186].

The synaptic crossing of the second-order sensory neurons of the TGN carrying touch discrimination, vibration, and proprioception occurs immediately in the mesencephalon to join the ventral trigeminal lemniscus (medial lemniscus) and to then synapse on third-order neurons in the ventral posterolateral (VPL) thalamus [186]. In contrast, pain and temperature information, as mentioned, must travel distally to the spinal trigeminal nucleus in the upper cervical cord, where it converges with central cervical neurons; it then ascends contralaterally as the spinal lemniscus tract to the level of the pontine nuclei, where it will abut and travel just posteriorly to the ventral trigeminal lemniscus tract to innervate primarily into the ventro-posteromedial (VPM) thalamus before ascending to higher cortical regions [186].

The TGN’s large sensory ganglia functions as the significant source or “central hub” of sensory information from the face, mouth, ears, nose, blood vessels, and cranial dura, thus playing a critical role in our capacity for self and environmental processing [187]. See [173,186,187] for a complete list of TGN anatomical innervations. However, the innervation of the nasal region by the TGN and its central consequences are primary to the current discussion. Specifically, a branch of V1 pushes through the cribriform plate anteriorly. It innervates the nasal cavity, the integument (mucosal lining) of the nose, the frontal sinuses, and portions of the eyeball [70], while a branch of V2, the nasopalatine nerve, supplies the posterior turbinates, and the ethmoidal branch serves the anterior portions [70]. Additionally, V2 innervates the nasal mucosa, the other three nasal sinuses, portions of the eye, the pharynx, and the meninges, relaying somatosensory information [185].

As mentioned previously, the trigeminal nerve endings are considered free nerve endings because they lack an epithelial covering [188]. These nerve endings sit just a few micrometers [189] below the level of the tight junctions within the nasal mucosa and exhibit a copious number of heterogeneous nociceptive ion channels responsible for nociceptive processing [188,189]. It has been suggested that the trigeminal system is then deficient in its role in processing nasal irritants as the exposed nerve endings are inaccessible below the tight junctions [190]. However, nestled in between the nasal mucosal cells, the goblet cells, and the basal cells are specialized, highly conserved cells called solitary chemosensory cells (SCCs); these cells form direct receptive contact within the nasal mucosa through their apical extensions, while also directly, and at times completely, enveloping of the trigeminal nerve endings below [188,191]. First identified in the fish in 1960 by Mary Whitear, later in mammals by Finger et al. in 2003, and now in the human nasal epithelium [188], these chemoresponsive cells (chemoreceptors) respond to diverse irritants, bacteria, and viruses by activating molecular markers, such as transient receptor potential channel 5 (TRPM5), T1Rs, and T2Rs (taste receptors), which utilize similar downstream signaling effectors like G protein α-gustducin and phospholipase C beta 2 (PLCB2), consequently synapsing onto the polymodal nociceptive fibers of the ethmoidal branch of the trigeminal nerve [191].

The anatomical placement of the SCCs is logical. High concentrations are found in the anterior portion of the nasal cavity and within the inferior turbinates, where the initial influx of air and airflow is focused [192]. Hence, the location is ideal and critical for first-line defense against the inhalation of harmful toxins where activation of trigeminal-based evolutionary (protective) reflexes, such as coughing, sneezing, and respiratory rate changes, will favor the preservation of homeostasis and survival [191]. In fact, the seminal work by Finger et al. showed close to apneic breathing rates in anesthetized rats within seconds of administration of a known trigeminal irritant, cycloheximide [191].

The trigeminal nerve can receive and process various types of mechanical, sensory, nociceptive, and temperature information. Histologically, the trigeminal nerve houses large-diameter fibers for transmitting proprioceptive information, fast-conducting large mechanoreceptor fibers (Aβ-fibers) for processing light touch and vibration, unmyelinated C fibers responding to chemical and thermal activity, and, finally, the Aδ fibers, which are small-diameter, fast, myelinated fibers processing cold stimuli and sharp pain [188,193]. Therefore, activation of the trigeminal nerve leads to various cortical sensory experiences, such as burning, stinging, itching, tickling, warming, cooling, and pain [193]. Additionally, the activation of the previously mentioned SCCs from fluctuations in humidity, pressure, temperature, pH, and potentially harmful irritants, including viruses, can initiate complex chemical signaling cascades, in part involving transient receptor potential (TRP) channels [193]. Extra-nasally, primarily through the Vagal and trigeminal nerves, they contribute to the sensory experience from the lungs, kidneys, gastrointestinal tract, skin, liver, heart, cornea, and spinal nerves [194]. These multimodal ion channel *sensors*, first discovered in 1969, can respond to chemical and physical stimuli [194]. The name refers to the first observation and discovery of the transient response of the Drosophila to light stimulation by Cosens et al., yet they were later coined TRP channels by Minke et al. 1975 [194]. Fast forward to 2021, when the Nobel Prize was awarded for identifying that a subgroup, TRPV1, transmits pain and temperature sensations [194]. Interestingly, the TRPV1 receptor found throughout the trigeminal nerve is also activated by capsaicin and carbon dioxide [183]. The subfamily TRPA1 receptor, which responds to thymol, allicin, gingerol, mustard oil, and cinnamaldehyde [195], is often located within the same sensory nerves as the TRPV1 receptor, like the nociceptor fibers found in the trigeminal nerve, and prolonged activation has been linked to increased seizure activity [183,194]. Also, a part of this 28 “member” group located on the trigeminal nerve are the menthol- and cool air-responsive transient receptor potential melastatin-8 (TRPM8) proteins [196]. As a group, TRPs are considered “cellular sensors.” As such, they fittingly specialize in receiving, integrating, and transforming a wide range of external environmental stimuli, such as cold, heat, humidity, viruses, voltage, mechanical force and stretch, pH, and natural compounds like herbs, oils, and more, into essential neurological functions of vision, taste, and smell, as well as a plethora of sensory sensations like coolness, burning, and stinging [195]. Additionally, Liu et al. suggest an interesting universal mechanism through distortional and conformational changes in the plasma membrane, equating them to mechanosensitive channels even when the original stimulus is not mechanical [197]. The TRP channels’ dynamic and varying polymodal activity makes them uniquely capable of transforming an impermeable (epithelial) membrane into a permeable one [198]. For more on signaling pathways, TRP categorization, and a chronological discovery description, see [194,195,198,199,200].

## 9. Exhaled Breath

Interest in the components of human exhaled breath (EB) for its medical value dates back to 400 B.C.E. [201]. Since Hippocrates, people have associated diabetes with sweet or fruity breath and kidney diseases with a fish-like odor [202,203]. Currently, breath analysis research is increasing in popularity as it is of non-invasive, potentially widespread diagnostic value for lung cancer and lung-related disease [204], colorectal cancer, diabetes, metabolic variations [204], and COVID-19 [203].

Exhaled Breath Condensate (EBC) is considered a “biofluid,” a condensed water vapor [203]. Therefore, it represents an aqueous environment referred to in the literature as airway surface liquid (ASL), epithelial lining fluid (ELF), respiratory fluid (RF), or alveolar lining fluid (ALF) and is said to be representative of the central airway constituents [204,205]. The components of EBC are voluminous and complex, creating a mixture of volatile and non-volatile compounds (VOCs and nVOCs) [206], hormones, fatty acids, inflammatory markers, salts, proteins, viruses, and pathogens [202,203,205]. Please see [202,203,205] for a detailed discussion on EBCs. EB was first thought to “contain no less than 200 VOCs” by Linus Pauling in 1971 using gas chromatography [207]; it has now been demonstrated to contain over 3500 VOCs [202]. EB also contains inert gases (0.9%), water vapor, inorganic VOCs such as nitric oxide and ammonia, and organic VOCs like acetone, ethanol, and methane [202]. However, the foremost gases in human EB are “nitrogen (78.04%), oxygen (16.0%), carbon dioxide (4–5%), and hydrogen (5%)” [202]. A healthy individual’s average amount of EB is about 500 mL, of which 150 mL is dead air space [202]. Dead air space is the air volume that acts as the conducting path in the upper airway and does not participate in gas exchange, yet does dilute and affect the markers found in EBC [202,203]. Therefore, the end-tidal breath, the last bit of exhaled breath, is the closest to the alveolar breath composition [202]. Because the volume of soluble gases (i.e., CO_2_) is diluted by increased blood flow and by reabsorption into the airway’s mucus membrane as it travels through the airway’s path, it has been suggested that holding one’s breath for 10 s before collection would lead to higher concentrations of those gases in the EB [202]. Please review [208] on the Human Breathomics Database (HBDB).

Other factors to consider relative to EB are variations in individual component concentrations, like pH, temperature, and relative humidity. The concentration levels of the multitude of elements in EB, like VOCs, will vary from person to person based on environment (exogenous), age, gender, metabolism (endogenous) [209], smoking behavior, BMI [210], and numerous disease states, as they reflect the somewhat unique internal metabolic cascades occurring in the body [211]. However, the percentages of the leading gases found in EB appear to be relatively similar across subjects [209]. The pH of the ALF, for example, has been reported to be about 6.54 when the partial pressure of CO_2_ is measured at 5.33 kPA (alveolar pressure of _p_CO_2_), expressing an inverse relationship [204,212]. The pH of the EB in human studies can also vary slightly with age and the health status of the individual, yet the broad consensus regarding the pH of exhaled breath in healthy persons is that it is held within a narrow range, favoring slight alkalinity [213].

Furthermore, we do know that individual and environmental variations can and do occur. For example, in acute asthmatics, the pH is lower than that of healthy controls yet remains similar in smokers and exacerbated COPD individuals [214]. A recent study by Rama and colleagues found a higher and lower EBC pH in children with asthma [215]. However, the higher pH cohort was theorized to indicate a particular asthmatic phenotype. Still, the overall findings were that asthmatics had a more acidic pH, even considering the numerous environmental and physiological variances, such as recent exercise, food ingestion, and ecological (room) metrics [215]. When evaluating EBC pH, temperature and relative humidity of the environment must also be considered. For instance, the average EB temperature has been reported to range from 34–35 °C; the relative humidity is higher in females, yet unpredictable and geographically independent, ranging from 41–100% [216]. Carpagnano’s group, in a large study, concluded that the exhaled breath temperature (EBT) was 30.459 °C ± 2.955 °C in Caucasian healthy nonsmokers [217]. Please review [217] for a discussion on pertinent factors affecting EBT.

A study by Salati and colleagues on the effect of respirator masks and CO_2_ inhalation with the use of computational fluid dynamics (CFDs) showed that after exhalation, the nasal cavity temperature was 36 °C in the superior turbinate area and 34 °C in the middle turbinate region. This temperature discrepancy intranasally illustrates the differences in airflow dynamics; as airflow is diminished, temperatures rise, as in the superior turbinates. In contrast, greater airflow exists in the anterior vestibule and the middle and inferior turbinates, so temperatures decrease slightly. The authors showed that water vapor, temperature, and CO_2_ all increase within the mask after one breath cycle and that CO_2_ concentrations increase up to five-fold over ambient air [218]. The subsequent inhaled air from the mask interacts with the anterior nasal mucosa and is perceived as cool air, compared to the heated mucosal lining; this activity is then communicated to the CNS via the trigeminal cool receptors, TRPM8, which are numerous in the nasal mucosa [218]. Simultaneously, the inhaled air is warmed as it moves through the middle and inferior turbinates, where the bulk of the inhaled air travels [218]. The stimulation of these receptors is so influential that studies show a reduction in inspiratory drive and alleviation of breathlessness upon activation [196]. These cool receptors, once activated, along with all other TGN somatosensory information, are quickly relayed through the TGN ganglion to the midbrain, pons, medulla, and spinal nucleus for initial integration into the ventral posteromedial thalamic nuclei [196]. The information continues to be disseminated to multiple brain areas, like the postcentral gyrus, anterior cingulate, insula, and somatosensory regions [196].

Investigational research on EBCs and their various constituents as potential biomarkers and indicators of health and disease are still being tested and questioned. In particular, one of these challenges is the precise measurement of EBC’s pH. To illustrate, it is known that the airway buffering concentrations of NH_3_/NH_4_^+^ (ammonia/ammonium) and CO_2_/HCO_3_^−^ (carbon dioxide and bicarbonate) can significantly affect the EBC’s pH [215]. Accurately calculating these concentrations is problematic as they can be affected by the type of measuring device used, environmental conditions, and the physiological parameters associated with the individual, such as smoking and drinking [214]. Please note an extensive review is beyond the scope of this paper. However, we want to acknowledge the apparent diversity of the studies and findings concerning EBC’s constituents and concentrations, elucidate the scope and complexity of the topic, and illustrate emerging themes in the study of human exhaled breath and its relevance and role in human physiology and its potential for therapeutic application. Regarding the scope of experimental techniques utilized for assessing EBCs, please see [219] for an excellent review.

## 10. CO_2_ Diffusion, Molecular Qualities, and Physiological Characteristics

A partition coefficient defines the concentration ratio of a “compound at equilibrium when dispersed between two immiscible phases,” which is temperature dependent [220]. The partition coefficient of a substance, whether a drug like Diazepam or CO_2_, determines its ability to and rate of transfer through a membrane, like across a biomembrane such as a cell wall [68]. The biological gas CO_2_ uniquely expresses multifaceted phase behaviors, as it can exist as a solute, liquid, or gas [221]. It is highly lipophilic but also hydrophilic, with a high biological cell permeability value [222]. In this regard, CO_2_ is unique from other gases like O_2_ and N_2_ because it has 20–50 times greater solubility in water and up to 20 times higher solubility in oil [222]. CO_2_’s increased solubility and, hence, diffusion is due to its linear molecular bond structure, its slight electronegativity (dipole), and its resultant dipole–dipole interaction with water, which is in direct contrast to oxygen’s molecular nonlinearity and non-polarity. The concept that gases diffuse quickly into cell membranes was first fostered by Ernest Overton in 1895; by the early 21st century, it was considered a truism, as evidenced by its place in chemical textbooks [222]. Yet, in 1976, Finkelstein raised the issue that a compound’s membrane permeability ratio is determined not only by its diffusion coefficient but also by the barrier or resistance it encounters within the membrane itself, such as cholesterol, which will potentially alter concentration levels dynamically based on the physiological conditions encountered [222,223]. For instance, the human red blood cell contains 45% membrane cholesterol and, at face value, would only allow for a CO_2_ permeability rate of 0.015 cm/s. Yet, in actuality, a permeability rate of 0.15 cm/s ensues because of two identified membrane proteins, Aquaporin 1(AQP1) and RhAG, constituting gas channels occurring in significant quantities on RBCs and on kidney tubules’ apical membranes that directly influence the diffusibility of CO_2_ in mammalian cells [223]. This 10× CO_2_ permeability rate increase appears to be a species-specific evolutionary adaptation highlighting the importance of CO_2_’s permeability and, correspondingly, an organism’s oxygen consumption [223]. Aquaporins are a group of “water channels” that facilitate the transport of water through cellular membranes (bio-membranes) [224]. Thirteen mammalian Aquaporins are now recognized to participate in diverse cellular transport assists, cell migration, cell adhesion, and cell proliferation, depending on their particular permeability properties [224]. Pertaining to the current discussion, Aquaporins 1–4, 5, 7, and 11 are spread throughout the human nasal cavity [224]. AQP4s are located in the nasal respiratory epithelium.

See [225] for a molecular and historical rationale. Like most topics in science, debate exists. Please review [226] for an older yet concise summary of discussion points about CO_2_’s role and permeability. Additionally, see [223] for a look into the differences between fish, mice, plants, and humans and the potential role of gas channels. Finally, please review [227] for a review of cholesterol’s unique role in human RBC cell membrane permeability to CO_2_. Another element to consider in the apparent, rapid, and unique diffusion rate of CO_2_ is the enzyme Carbonic Anhydrase (CA), a zinc-containing metalloenzyme that promotes the reversible conversion (hydration and dehydration) of carbon dioxide and bicarbonate [228]. Sixteen human isoforms have been identified and are located in RBCs, kidneys, lungs, ovaries, nasal mucosa, and various brain regions, like the hippocampus [228]. See [228]. CA plays a vital role in many biological pathways and processes, including pH maintenance, glucose and lipid release (or genesis), augmented signal activity, and long-term potentiation in memory consolidation [229]. Consequently, many carbonic anhydrase inhibitors (CAIs) are now manufactured for clinical applications and include, but are not limited to, the treatment of epilepsy, retinopathy, anemia, obesity, and cancer [228]. Recently, CAIs have been investigated for the modulation of inflammation, neuropathic pain, glaucoma, edematous conditions, and as an anti-infective agent [230]. Furthermore, CA activators (CAAs) are now considered seriously in treating neurodegeneration and aging [230]. Interestingly, CA’s chemical structure features a zinc-containing active pocket specific for CO_2_ docking, which readily promotes the conversion of CO_2_ molecules to bicarbonate ions [231]. Thus, proving to be a potent CO_2_ converter, its strong utilization potential is now actively recognized in environmental applications [231]. However, considering the current discussion, CAs seem to play an essential role in the diffusion rate of CO_2_ across the cell membrane by altering dynamically and continuously CO_2_’s concentration gradient throughout the entire cell environment; this is in contrast to the previously mentioned gas channels that strive to increase cell functional permeability [225].

In 1978, Sakakibara and colleagues were the first to identify that the nasal sensory epithelium in bullfrogs mediated reflexive respiration through local CO_2_ receptors, and it was not mediated by the larynx or glottis as previously thought by Smyth et al. [232,233]. Furthermore, using a denervation model, they demonstrated that nasal innervation by the olfactory nerve and the trigeminal nerve conveys CO_2_ concentrations centrally. Interestingly, while both caused initial apnea or diminished respiration in response to elevated CO_2_ concentrations, the trigeminal response to activation resulted in a brief “filling-like respiration” distinct from the olfactory response [232]. This suggests that while both cranial nerves are shown to be involved with reflexive respiration, the subsequent activation leads to divergent central reactions. Note that it was Smyth et al., in their 1939 paper, who postulated that the medullary region was, in fact, sensitive to elevated CO_2_ and diminished O_2_ levels [233]. As reported by [233], earlier work by Von Budenbrock and others showed that 2–10% CO_2_ resulted in hyperpnea and increased respiration in frogs [233]. Thus, CO_2_ can cause activation or inhibition of the central and peripheral chemosensory receptors depending on the species, for example, frog, rat, fish, bird, reptile, and human, the current integration of the central respiratory network, and whether the CO_2_ change occurs systemically or in the upper respiratory tract [234]. Additionally, the concentration and location of CAs, the anatomical route of administration, and the species’ evolutionary nature are determinants for a species-specific ventilatory reaction to elevated CO_2_ [234]. For instance, some reptiles will slow respiration in elevated environmental CO2 situations until higher O_2_ concentrations are perceived [234]. Please review [234,235,236] for more on evolutionary and species-specific CO_2_ chemosensory dynamics.

## 11. Exhaled Breath Rate and Inhalation Airflow Velocity and Consequences

In the early 2000s with SARS (severe acute respiratory syndrome, 2003), H1N1-(novel swine-origin influenza A, 2009), consideration and interest in the mechanism of airborne transmission of viruses began to take center stage [237]. This trend has increased even more since the arrival of COVID-19. As a result, increased and expanded investigations into the components of human exhaled breath, such as rate, velocity, distance traveled, and jet stream elements, essentially the components of fluid mechanics, have now become substantially more prominent in scientific arenas that are concerned with viral transmission and societal health and well-being [238]. Per this discussion, there are two relevant aspects of human exhaled breath, which are flow rate, defined as the fluid or gas volume passing per unit of time within a specific region or area, like a channel or pipe, and velocity, which is quantified as the distance traveled in each time frame. Bourrianne et al. measured expiratory flow rates utilizing warm CO_2_ as a marker in humans with and without face masks [239]. They showed that exhaled air forms jet streams capable of “reaching several tens of centimeters to 1 m within one second” of exhalation in unmasked individuals [239]. Additionally, they recorded variances in flow rate in line with the type of exhaled air. For example, soft nasal exhalation reached a maximum flow rate of 15 L/min (0.25 L/s), expired air through a relaxed open mouth reached 40 L/min (0.67 L/s), and heavy respiration and active blowing were noted at 200 L/min (3.3 L/s) [239].

Li et al., while studying short-range airborne transmission indoors, estimated light, moderate, and heavy activity breath flow rates to be 0.02 L/s, 0.3 L/s, and 0.6 L/s, respectively [240]. Abkarain et al., studying asymptomatic virus spread during speech and regular breathing, found that exhaled air from subjects during normal breathing reached 0.5 to 1 m within 1 s and that the volumetric airflow rates were 0.2 to 0.7 L/s for normal breathing over a 3–5 s breath cycle [238]. These findings appear in the range of an older study showing a peak flow rate of 0.7 L/s during natural breathing [241]. The human breath cycle in adults is 10–15 cycles per minute, while in children, a higher rate of 18–20 is considered normal [242]. While speaking, Chao et al. recorded an average airflow velocity to be 3.9 m/s, whereas Kwon et al. measured 4.07 m/s and 2.31 m/s airflow velocities for males and females, respectively [237,243]. In contrast, Han et al. measured a maximum instantaneous velocity of 6.25 m/s [244]. The latter report may match Giovanni’s group, which found that a long exhalation preceded by a long inspiration produced the greatest velocities compared to speech and vocal exercises [245].

We recognize, as others do, that comparing and validating the distinctive components of human exhaled breath is burdened by its inherent transient, situational, and environmental variability and further confounded by several individualistic factors, such as height, BMI, gender, and the presence of diseases like COPD and asthma, altogether creating a challenging landscape [237,241]. However, the range of 0.2–0.7 L/s seems reasonable based on the literature reviewed. Please see [246] for a description of the potential gender differences in air velocity and flow.

In order to understand the EBM, we need to consider not only the flow rate and constituents of the expired air but also inhalation flow rates (velocity), anatomical nasal variations, and physiological and volumetric consequences both peripherally and centrally. To gain insight into the therapeutic EBM for a seizure disorder, we need to understand the breadth and depth of the dynamic interactions of inhaled air along the entire nasal and respiratory route under healthy, normal breathing conditions in humans, so that we may attain a greater understanding of pathological and physiological deviations, along with appropriate drug applications [247]. A complete picture is still evolving, and many questions remain, such as what constitutes a normal intranasal airflow. What is the effect of the nasal cycle on health and disease? What is the best model to evaluate biological normality and intranasal drug delivery? How accurate and representative are the nasal modeling studies? How do various nasal morphologies, as identified by age, sex, and race, contribute to nasal dynamics in health and disease? As a result, investigators from diverse disciplines like toxicology, otolaryngology, neurology, and epidemiology, to name a few, along with advancing technologies like 3D modeling, UV mapping, and advanced CFDs, are striving to this end. Zhang et al. confirmed this trend using scientometric methods to comprehensively review non-invasive respiratory drug therapies over the last 20 years. Their group reported that from 1998 to 2020, publications grew from less than 100 to 336 and included investigations on nasal drug delivery for CNS diseases, PD, AD, depression, tumors, and epilepsy [248]. The advantages of intranasal delivery are bypassing the blood–brain barrier, direct brain delivery via olfactory neurons, indirect delivery through the TGN, systemic delivery through the rich nasal vascularization, high permeability, and surface area [248]. Moreover, they are convenient, quickly accessible, have the potential for self-administration, and are not modulated by gastrointestinal and haptic metabolism [248].

The underlying physics of intranasal fluid dynamics and nose-to-brain delivery in healthy non-diseased persons, along with investigations into the physiological and biological aspects of numerous drug preparations and their local and central consequences, from chronic rhinitis to the treatment of Parkinson’s and Alzheimer’s disease, are taking place. Focusing on the physiological characteristics of the nose is necessary to create effective nasal solutions for treating chronic rhinitis, for example. To begin, we know there are immediate and dynamic interactions with the inhaled air along the entire nasal and respiratory route under healthy, normal breathing conditions in humans, and these need to be considered for a deeper understanding of pathological and physiological deviations [247]. We know that incoming air under normal conditions produces mechanical and physical stress on the local nasal epithelial mucosa, known as wall shear stress (WSS) [247]. It mainly occurs in the anterior nasal chamber and at the entry of the middle meatus passage [247]. Small human preliminary studies on nasal irrigation parameters confirm that the WSS is more significant with larger volumes occurring in the vestibule, anterior respiratory epithelium, and middle turbinate, with sequential degradation as the solution moves posteriorly through the nasal cavity [249]. Also, with an increased solution volume, there is a correlated increase in irrigation volume but a reduced local residual active volume [249]. A higher squeeze force of the solution results in superior penetration to the paranasal sinuses and the olfactory epithelium, yet a slower squeeze force leads to a lengthier irrigation effect [249]. In line with the former, Shrestha showed that a 45-degree backward head tilt increased olfactory epithelium coverage over other head tilts [250].

Similarly, a larger quantity of solution allows volumetric overflow into the sinus ostia, providing penetration into the sinuses, which is most significant in the ethmoidal and maxillary sinuses [251]. Shrestha et al. also showed that the WSS in the paranasal sinuses varied significantly with head-tilt positions, and the maximum local WSS was the highest in the ethmoid sinus when compared to the other sinuses [250]. Likewise, increased olfactory penetration and contralateral paranasal sinus effect were observed with a 45-degree backward head tilt [250]. Interestingly, a 45-degree right head tilt (not left) displayed greater exposure (second to the backward head tilt) with left nasal irrigation in the contralateral paranasal sinuses-maxillary, ethmoidal, and frontal [250]. For adequate liquid (jet) flow, velocity and mass are also needed [251]; thus, the nasal cycle must also be anticipated, as the stage of the NS affects volume and flow rate and deposition of drugs delivered intranasally and, therefore, the physiological treatment effectiveness [252].

Regarding the above-mentioned paranasal sinus findings and their relevance to the current topic, recall that the paranasal sinuses are covered with pseudostratified ciliated columnar epithelium and mirror the same additional cells and sensory and autonomic nerve supply as the nasal epithelium [253]. Likewise, the sinuses are innervated by branches of V1 and V2 of the trigeminal nerve [167] and thereby symbiotically serviceable for the same tasks as the nose: humidification, temperature control, enhanced mucosal surface area, olfactory processing, and serum _P_O_2_ and _P_CO_2_ safeguarding [253]. Debate still exists regarding the exact nature and function of the paranasal sinuses. Yet, recently, an evolutionary slant has been proposed, in short, based on bipedalism and the physical forces required for fluid management on land [253].

We know there are simultaneous and dynamic interactions of inhaled air along the entire nasal and respiratory route under healthy, normal breathing conditions in humans, and these aspects help us understand drug delivery considerations [247]. Research investigating intranasal drug delivery must be carefully considered as there are essential and specific differences between humans’ and animals’ nasal anatomies. For example, rats have almost four times the amount of olfactory epithelium (3350 mm^2^/cm^3^ vs. 820 mm^2^/cm^3^) of humans [254]. The respiratory epithelium represents only 50% of their nasal cavity, in contrast to the 80–90% coverage of humans [255]. In humans, moving anteriorly to posteriorly, the vestibule, located at the nasal entry, is estimated to be 0.6 cm^2^, the respiratory epithelium is the largest and most vascularized intranasal region measuring 150 cm^2^, and, finally, the olfactory region covers about 10 cm^2^ of the nasal region [255]. These principal regions in the nose are concerned with various aspects of the incoming air, like airflow parameters, its constituents, humidification, pH and temperature control, and the absorption of molecules, and vary to some extent between species [254,255]. Sasaki et al. report that because of the rats’ larger olfactory region, drug absorption analyses can over-estimate drug deposition and efficacy and that the nasal apparatus of cynomolgus monkey is more analogous to humans; however, studies using non-human primates are significantly fewer [256]. Additionally, the accessibility of the olfactory region differs among rats and humans because the nose is narrower and longer and a septal oval window is absent in the former [255]. Anatomical variances between species should be considered as well. See [255]. Differences also exist among human adults and children. The maxillary sinus, for example, continues to grow downwards past the nasal floor by the age of 12 and is not fully mature until around 20 when the permanent teeth are fully established; at this time, the sinus will consolidate in size but remains larger in men than in women [253].

## 12. Nasal Breathing, History, and Central Nervous System Consequences

The earliest recorded yoga theory is the Upanishad philosophy of the Indus-Sarasvati civilization in 2500 B.C.E. [257]. It appears this civilization, because of their settlement along the river and the resulting new era of agricultural life and robust food supply, were able to shift their nomadic life centricity and priori of survival to one of the studies of life itself and began to practice physical forms of yoga and meditation [257]. The incorporation of Vedic Brahmanism in 1500 B.C.E. focused on ritual worship, chanting, concentration, and breath control [258]. The latter incorporates the earliest documented concept and interest in nasal dominance and its implied effects on the human experience and brain function [259,260]. The application of the Yogi crutch (Danda) to the axilla is alleged to initiate a reflex from the myofascial and nerve tissues and the skin of the axilla that is then transmitted to the spinal sympathetic [261] nerve fibers from the associated spinal segments related to the shoulder region [260]. More recently, nasal airflow has been recognized to be influenced by body posture, where the nasal patency of the upward-facing nostril will be increased while in the side-lying recumbent position [179].

In Swara yoga, the patency of the left nostril is Ida, and the patency of the right nostril is Pingla [262]. It was believed that unilateral left nostril breathing (ULNB) would positively affect activities requiring concentration and focused stationary work like spiritual endeavors because of its cooling effect, which yogic thought believes is connected to the right brain [263], and would predominantly affect the parasympathetic limb of the autonomic nervous system [264]. In contrast, the more heat-generating right-sided nostril breathing (URNB) is more advantageous for physical undertakings like chanting and hunting [262], influencing sympathetic tone and arousal [264], and is thought to be interlinked with the left brain [263].

The practice of intentionally controlling one’s breath to alter one’s psychophysiological state in the Science of Yoga is called Pranayama [262]. To understand and compare the potential consequences of Pranayama in the literature, we must define and clarify the different techniques. Two of the techniques are ULNB, referred to as Chandra Nadisuddhi pranayama or Chandra anuloma villoma pranayama, and URNB, known as siryanadi pranayama or surya anuloma villoma pranayama [265]. Equally, they are outlined as one complete cycle of breath involving an inhale and exhale alongside obstruction of one nostril, with varied breath retention and pacing inserted at the beginning and end of the cycle [262,266]. In both, occlusion of the nostrils usually uses the nasika mudra of the right hand, in which the ring finger occludes the outside left nostril, the thumb occludes the outside of the right nostril, and the index and middle finger are typically folded inwards on the palm, or alternatively, placed on the third eye [265]. In 2017, a systematic review was conducted on the hypotensive effects of yogic breathing and concluded even amongst the wide variety of study designs and yogic breaths practiced, that breathing practices with lengthier rhythmic pacing and ULNB had more advantageous autonomic outcomes [266]. In conjunction, a study measuring and describing the human nasal cycle by Kahana-Zweig showed slower respiration resulted in a more robust nasal cycle where airflow was measurably dominant in one nostril over the other, which was independent of the wake–sleep cycle [179]. The nasal cycle is an expression of autonomic status and functionality and is consequently representatively interconnected to asymmetries in cortical functions [179].

In 1963, Kleitman theorized and characterized the existence of a basic resting-activity cycle (BRAC), which occurs throughout the human sleep cycle, expressed outwardly as periods of rapid eye movement (REM) alternating out of phase with non-rapid eye movements (NREM) [267,268]. He believed it represented rhythmic brain activity fluctuations that predictably transpired in 90-min cycles [267]. Since then, many studies have demonstrated that non-circadian biological cycles exist and are known as ultradian cycles. As mentioned above, an ultradian rhythm (UR) or cycle is a preserved, endogenous, self-generating biological rhythm that reflects and influences a species’ behavior and physiology, like breathing and heart rhythms [269], locomotion, feeding, hormone release, sleep cycle REM and nREM [270], body temperature, blood flow, oxygen consumption, carbon dioxide production, blood pressure [268], and the nasal cycle [271]. These cycles regularly act, lasting from milliseconds to hours, and proceed both independently and superimposed upon other circadian rhythms [270]. Yet, URs are also known as the “outlaws of biological rhythms,” confounding scientists because of their, at times, nimble and subtle variations in frequency, timing, and interval length [268]. In part, because of these factors, the underlying triggers or mechanisms explaining these rhythms are still unidentified. Yet, the midbrain, hypothalamus [270], medulla [271], and hippocampus [268] are the currently proposed neural substrates capable of acting as central (master) oscillators supervising theses rhythms. In general, these rhythmic systems can be tied to the adaptability, arousal, and, thus, survivability of the organism [268]. The ultradian rhythms should not alone be thought of as acting upon the organism in a constrained anatomical corporeal paradigm, but rather a global synchronization of many physiological subsystems communicating and reacting to the current biological status of the organism. To illustrate, a change in temperature by a few tenths of a degree will prompt an increase in cellular metabolism within every cell exposed to that change [268].

The subject of the nasal cycle, nasal airflow asymmetries, their relationship to human physical and cognitive performance, and the activation of specific brain (hemispheric) regions have been of great interest to many researchers past and present. The nasal cycle fits well into Kleitman’s BRAC organization of alternating, reciprocal, self-initiating, spontaneous, non-circadian biological rhythms [272,273]. The deep nasal submucosa is richly innervated with sympathetic and parasympathetic fibers, allowing for alternating unilateral congestion and decongestion throughout a 24 h period [274]. Therefore, one side will experience more significant airflow under the UR’s pacing of increased sympathetic tone, while the contralateral side, under increased parasympathetic tone, will demonstrate reduced flow. The former indicates the active phase or dominant side, and the latter represents the rest phase [275]. Please note that four types of nasal cycles are described in the literature; please review [177] for details. Nasal patency is variable, individualistic, and under autonomic control. The nasal cycle regulates the airflow throughout the day, presumably to counteract the vulnerability of continuously open passageways to the outside world [276]. The goal is protection from various pathogens while maintaining adequate respiratory functions by increasing nitric oxide accumulation, increasing mucosal water concentration, boosting mucosal pumping mechanisms for waste removal, and other asymmetrical activities of the autonomic nervous system [177]. Amazingly, a German physician, Richard Kayser, in 1889, was the first to describe this lateralization as changes in “vasomotor tone throughout the periphery on two sides of the body” [177,274].

In this context, we have evolved primarily from symmetrical organisms to asymmetrical lateralized ones, laying the groundwork for an enhanced organizational development that serves enhanced cortical and physical functionalities. With knowledge of Kleitman’s work, Shannahoff-Khalsa explores further the lateralization and partial autonomy of anatomical bilateral structures and the unique adaptive unilateral ability of the ANS’ rhythmic operations [274]. We encourage the reader to review [274] for a historical perspective and a reminder that the ANS and CNS are complex and, at times, non-linear. Around the mid-to-late-20th century, numerous (animal and human) studies reported observations of the nasal cycle and the dynamic ANS’ reciprocal expression in the nose and anatomical correlates, such as glandular secretions, lung inflation, and pupillary changes [274]. In 1939, Samzelius-Lejdstrom observed in a large cohort that unilateral lung inflation on the ipsilateral side of forced unilateral nasal breath was significantly more prominent; this unilateral pulmonary nasal reflex was similarly witnessed in diseased states like tuberculosis [274]. Around the same time, and into the late 90s, it was found that lateral recumbency (and yogic crutch application) affected nostril patency with similar effects as could be elicited through applied mechanical pressure around the fifth intercostal space in the axilla. This resulted in demonstrable changes in the nasal cycle thought to be as a result of increased sympathetic tone on the contralateral side [274]. Davies and Eccles suggested that it was explicitly the skin receptors within the axilla corresponding to the involved thoracic segments and preganglionic sympathetic nerves on their way to the superior cervical ganglion before depositing into the vasculature within nasal mucosa that were answerable to these observations [277]. Similarly, Frye and colleagues postulated that pressure receptors residing throughout the pelvic and pectoral girdles and underlying, deep receptors and connections to the parietal pleura and intercostal tissue anatomically explain the pulmonary nasal reflex [271]. A more contemporary pilot study by Sinha et al. identified that changes in pulmonary function tests were associated with nasal dominance at rest [278].

Again, there is a plethora of human and animal studies that demonstrate the lateralization of the ANS. These insights are relevant for our discussion here, as these studies have led to the understanding, while still incomplete, that the CNS, particularly hemispheric (regional) function, is hard-wired with the ANS (nasal dominance), which Werntz et al. first showed in 1980 via EEG recordings that showed changes in the contralateral hemisphere from the dominant nasal cycle [279]. More recently, dominant ULNB showed EEG signatures associated with the left inferior frontal and left parietal lobe, which was in contrast to the nondominant ULNB, which had more significant but diffuse effects in the posterior areas of the brain bilaterally [275].

## 13. Discussion

There is no doubt that safe, effective, and rapid rescue from seizures in PWE is needed. Regrettably, there is no current cure for seizures and epilepsy; there is only prevention and management [280]. Almost one-third of PWEs are estimated to be non-responsive to ASMs [248,280,281]. Likewise, TLE, the most common type of focal epilepsy, results in 40% of postsurgical persons reporting seizure activity at two years [282]. Reasons behind these shortfalls are thought to be ineffective drug targeting, loading, mechanism of action, genetic variations, or specific disease-related confounders. Adding to this is the fact that the drugs currently used were historically manufactured and developed based on the current but limited seizure models of the time [281]. Unfortunately, the past several decades have produced very few “new molecules” that have passed the approval process and translated into effective therapeutic agents for terminating seizures [283].

Importantly, rapid interventions are particularly warranted in status epilepticus, DRE, and acute repetitive seizures, known as seizure clusters, in which immediate medical intervention is needed [284]. In the long term, stopping a seizure in situ and diminishing the reoccurrence is crucial to prevent further occurrences, self-perpetuating plasticity, neurological damage, drug resistance, morbidity, and mortality [285]. As mentioned previously, traditional ASMs, like benzodiazepines, act quickly and are potent, yet require expert administration (IV, intramuscular, buccal, or rectal delivery) or hospitalization or are socially undesirable, thus delaying treatment [284]. As a result, there has been an investigational surge for safe, quick, and effective practical alternatives.

The nose has long been considered a treatment path for PWE, which is not difficult to appreciate as descriptions of olfactory auras were first described in 1889 by a woman reliably smelling “dirty burning stuff” before seizure onset. Please see [281] for review. Even before that, in 1881, the application of strong scents like ammonia or “dirty shoes” was prescribed for rapid seizure arrest, and, specifically, Sir William Gowers utilized nitrite of amyl. Interestingly, additional extraneous somatosensory “type” therapies, including vigorous massage of the hands and feet, focal limb ice water baths, swallowing salt, strong taste stimuli, pin pricking, and other unpleasant stimuli, were utilized for seizure arrest [286]. In 1991, William Frey II proposed the first patent for intranasal drug delivery, thereby starting us on today’s journey of nose-to-brain drug delivery exploration and utilization to treat PWE [70,255]. Also, see [284] for a list of intranasal drug formulations.

The link between the nose (olfaction) and the brain is evident, and the piriform cortex (PC), the chief substrate of the primary olfactory cortex receiving direct inputs from the olfactory bulb, is central to this link governing olfactory experiences [287]. The piriform cortex is a phylogenetically old three-layered structure recognized to participate in and perpetuate epileptogenic activity and, like the hippocampus, is vulnerable to excitotoxic harm and neuronal hyperexcitability [287]. The PC is now recognized as an initiator and potentiator of seizure activity [282]. In focal epilepsy, various olfaction functions like odor detection, discrimination, and memory recall are altered or compromised, and in some cases, heightened olfactory function occurs in the prodromal state [287,288]. Physical changes have been reported as well, such as olfactory bulb volume loss [289,290] and hippocampal, entorhinal cortex, and amygdala cortical loss [282]. The PC and the amygdala have dense interconnections and are referred to as the “PC–cortical amygdala area (PCA)” [291]. Pereira and colleagues showed damage to this region may be directly associated with DRE [291]. The mitral and tufted cells that reside within the olfactory bulb send the structural connections from the glomeruli, the olfactory bulb’s synaptic element [224], directly to various layers within the PC, and, from here, subsequent widespread cortical and subcortical distributions are evident [287]. Please see [282,287] for review.

Not only are there direct connections from the olfactory structures to the PC, but human and animal research confirm trigeminal stimulus-dependent activation of the PC, corroborating overlapping central integration and modulation of the olfactory and trigeminal intranasal systems in response to nasal stimuli [292]. CO_2_ has long been recognized as a pure trigeminal stimulant, especially in low concentrations. The work by Carlson et al. showed that CO_2_ elicits activation of CO_2_-sensitive neurons within the PC, and these neurons can encode for stimulus modality and alter temporal aspects of either an “onset or offset” activation pattern [292]. These findings align with the work completed by Lunardi et al., where temporal changes persisted after olfactory stimulus presentation, revealing the olfactory cortex’s dynamical responsiveness to environmental stimuli like CO_2_ [289]. One mechanism behind this transduction of intranasal CO_2_ occurs through TRPA1 channels located on the nasal trigeminal nerves and through olfactory sensory neurons [292]. Intra-and extracellular pH changes from CO_2_ concentration alterations modulate both. This intimate link between CO_2_ levels and pH may explain the temporal results found in the PC and the effect that CO_2_ and odors have on modulating seizure activity. As CO_2_ increases, pH decreases, and as the CO_2_ is “metabolized,” the pH rebounds back to homeostatic levels. The active and rapid rebound of cellular pH serves the critical need for pH homeostasis, and it is this ever-mutable rebound that elicits synaptic signaling strong enough to create temporal and “offset” displays of cortical activation [292] in response to intranasal CO_2_, thus underscoring CO_2_’s import.

We postulate that the Exchange Breathing Method (EBM) has a combinatorial effect on the rapid termination of in situ seizures. The entire nasal environment is richly innervated by afferent somatosensory nerve endings responsible for detecting and transmitting noxious and chemical (CO_2_ and pH), thermal (temperature differentials with the external environment), and mechanical (airflow) stimuli to the CNS for rapid evaluation and resultant changes in neural activity of the organism [293]. A large portion of the local trigeminal nerve endings within the nasal epithelium are unmyelinated, polymodal C-type fibers that express TRP channel molecular sensors, voltage-gated Na^+^, K^+^, and Ca^2+^, and ASICs [293]; all contributing to a depolarization cascade that ultimately reaches regions in the CNS known to be involved with seizure manifestation and maintenance. Aquaporin channels and surrounding epithelial solitary chemosensory cells broaden and promote this rapid and robust chemical processing and absorption intranasally. Mechanoreceptive information is carried by the A-beta fibers with some spillover onto the C-fibers for transmission [294]. These superficial, rapidly adapting mechanoreceptive nerve endings quickly transmit neuronal signaling along the trigeminal nerve to the trigeminal spinal nucleus, cross within the dorsal spinal cord, and synapse into the contralateral ventral posterior thalamus, with further propagation to previously mentioned cortical structures.

Furthermore, based on the findings of Salati et al., we can extrapolate that the exhaled breath of one person into another’s nasal cavity, albeit warm, is relatively cooler than the recipient’s internal nasal mucosa, thereby activating the TRP cool receptors of the trigeminal nerve [218]. This exhaled breath concurrently adds additional water vapor, higher concentrations of CO_2_ (up to five-fold), and an amplified airflow stream that subsequently activates local ion channels, aquaporins, pain (CO_2_), temperature receptors, and mechanoreceptors; this barrage of TGN mechanical, chemical, thermal, and somatosensory information are quickly relayed through the TGN ganglion to the midbrain, pons, medulla, and spinal nucleus for initial integration into the ventral posteromedial thalamic nuclei [196]. Brew and colleagues found that an airflow rate equal to or greater than 1.7 m/s distributed by a hand-held facial fan reduced perceived breathlessness post-submaximal exercise [295]. Based on the Beaufort wind scale, that speed is classified between light air and a light breeze and, perceptually, is felt as such on the face. Hence, the activation of TGN afferents from the nasal mucosa by airflow alone is postulated to reduce breathlessness by the direct activation of the insular cortex, amygdala, and anterior cingulate, which all contribute to respiratory, pain, and anxiety networks and seizures [295].

Further support is elicited for trigeminal afferents’ critical role in the intranasal environment, and seizures can be observed clinically. Trigeminal activity can be witnessed postictally as a nose-wipe maneuver typically ipsilateral to the seizure focus, which is described as an “ictal rhinostomal sensation” caused by increased amygdala autonomic activation, subsequently increasing nasal secretions [287]. Other “rhinostomal” sensations can occur that reflect sensorial experiences of odorants, like itching, tickling, prickling, or a sense of pressure, and are believed to be activation of the TGN [287]. Additionally, we know that nasal respiration is connected and synchronized to the neuronal activity within the amygdala, PC, and hippocampus, possibly signifying the inherent entrainment of the respiratory cycle in humans [148,282]. This intranasal respiration and subsequent cortical activity are reduced when respiration is shifted to mouth breathing [148]. Historically and presently, there is sufficient evidentiary substantiation of the direct interconnectedness between nasal physiology and function (trigeminal) and their cortical consequences related to seizures.

Airflow velocity rapidly changes as it moves through the different regions of the nasal cavity, likely resulting in instant stimulation of multiple nasal receptors to successfully regulate thermal conditions for maintaining the health of the nasal tissue [252]. The airflow velocity must be strong enough to pass through the vestibule’s internal nasal valve [252]. Studies show that the inhaled air accelerates as it passes through this constricted region [247]. Afterward, the airflow velocity naturally diminishes, presumably increasing the contact time between the incoming air and the increased cross-sectional area of the nasal mucosa; this allows for proper temperature, humidity control, and gas exchange [252]. We therefore postulate that one of the leading mechanical differences between the EBM and bag breathing (typically used for hyperventilatory states) is the force (velocity) of the provider’s exhaled breath; while albeit gentle, it is substantial enough to penetrate the vestibule and internal nasal valve to instantaneously affect the profuse trigeminal nerve fibers smartly placed in the respiratory epithelium. Recall cortical activation of all associated limbic structures occurs with nasal respiration (activation) [148]. We know that the incoming air under normal conditions creates local mucosal wall shear stress (WSS) that produces mechanical and physical stress, particularly in the anterior nasal chamber and at the entry of the middle meatus passage [247]. In intranasal drug studies, we have observed that a higher squeeze force of the nasal solution leads to superior penetration into the paranasal sinuses and the olfactory epithelium. However, a slower squeeze force leads to a lengthier irrigation effect [249]. WSS occurs in the nose and penetrates the paranasal sinuses concurrently. We can appreciate, then, the rapidity, coverage, and effect of broad cranial stimulation with nasal irrigation and increased airflow. All four paranasal sinuses are linked to the nasal vault; as an anatomical example, the maxillary sinus communicates directly with the middle meatus (turbinate) through the hiatus semilunaris [253]. Therefore, this barrage of mechanical stimulation travels very quickly throughout the nose and paranasal sinuses, richly innervated with two of the three branches of the trigeminal nerve for central integration, as discussed above. These parameters are ideally expressed in the EBM. The provider’s EB is of sufficient velocity to penetrate the internal nasal valve but slow enough to bathe the trigeminally laden respiratory epithelium and paranasal sinuses simultaneously.

In this regard, we want to acknowledge a few reports of nasal airflow mechanical activation through unilateral nostril hyperventilation increasing epileptic EEG irregularities [296,297]. Two studies by Servit et al. on animals and later in humans showed increased EEG activity in the rhinencephalon by applying positive-pressure rhythmic insufflation at a rate of 16–18 times per minute for four minutes. Three EEG patterns were identified and compared with oral hyperventilation, then subsequently compared after anesthetization of the nasal mucosal [297]. Anesthesia of the upper (superior) turbinate area resulted in temporary suppression of the epileptic EEG abnormalities. The authors concluded that the cause was a reflex arc between the cortical limbic structures and the superior nasal meatus [297]. A case study on a young woman with reflex epilepsy by Llik and colleagues reported that the smell of paint thinner consistently triggered epileptic events; this was confirmed with EEG performed congruently with inhalation of the paint thinner [298]. Bozorg et al. reported a breakthrough seizure event in a 60-year-old male’s post-temporal lobectomy after exposure to an essential oil mixture during a massage that contained rosemary (camphor). Camphor, along with sage, hyssop, and fennel, are known to induce neuronal hyperexcitability in both epileptic and nonepileptic persons, with children being more vulnerable [299]. See [299] for a review. Seizure exacerbation from external sensory stimuli is well-known, yet reports on olfactory reflex seizures are rare [289]. External sensory stimuli have the potential to be either inhibitory or provocative and may have an underlying temporal summation with habitual precipitation of particular stimuli [289]. A multicenter international study by Lunardi et al. reported both provocation and inhibition of epileptiform discharges (ED) in temporal lobe epilepsy (TLE) and idiopathic generalized epilepsy (IGE), with the latter experiencing more inhibitory effects from olfactory stimulation (OS) [289].

Interestingly, they found that the response to OS (essential oil, Ylang Ylang) continued to manifest and develop in the 15 min interval between administrations of the stimuli, showing an individualistic, “variable, dynamic or regression” of ED. As the authors state, this is a unique finding among all other sensory stimuli studied, in which the effect of the stimulus occurs within the stimuli pattern [289]. Furthermore, no evidence of olfactory reflex epilepsy was confirmed in this cohort of 134 subjects. These few studies that show the potential provocation of epileptiform activity through nasal airflow or olfactory stimulation reveal distinct differences from the EBM. The studies by Servit explored the effect of nasal hyperventilation techniques and not a slow exchange of exhaled breath. The later studies utilized and reported the impact of high, pungent, or persistent nasal stimulation-specific smells on ED. The exhaled breath of the potential assistant is absent from such activity. Therefore, we propose that the EBM is inherently safe and potentially effective in arresting seizures in situ.

In reference to the findings of “variable, dynamic or regression” of ED with OS in the Lunardi study, we would like to note a study by Chen et al. Their high-throughput sequencing mapping of the PC found a spatially organized olfactory cortex [300]. In contrast to the prevailing model that suggested the PC is an unorganized “tabula rasa” network, they show specific topographical and parallel streams of organized projections between the OB cells and the anterior and posterior PC, including matched projections to extra-PC structures, creating “triadic circuits” [300]. Like the ventral and dorsal visual streams, which process the “what” and “where” of visual field information, respectively, the PC streams process odor valence, perception, and action/location in different regions and at different speeds with varying decay rates throughout the PC [300]. The anterior “sniff-locked” responses for odor identity are fast compared to the slower build of navigational processing of the PPC, which potentially explains the persistent and varied ED after OS [300]. These findings highlight the importance and link between mechanical “sniff activities” (intake velocity), which increase odor identity and are rapidly processed and cross-referenced from both indirect and direct olfactory pathways [300] for central integration. Their findings reveal a direct and systematic link from the OB (mitral and tufted cells) to the PC and to extra-PC cortical targets, allowing for specific behavioral reactions [300]. Like the visual system, they suggest topographical, functional, and locational organizational connectivity, optimizing plastic learning comparable to other human sensory systems. Please review [300] for details.

While odorants can be recognized retronasally through the mouth, sniffing is crucial for odor identity and affects the olfactory bulb’s functional network [301]. Because the olfactory epithelium and bulb sit superiorly and posteriorly within the nasal cavity, sniffing can be seen as a means to enhance odorant flux to the olfactory bulb and receptors for rapid and accurate decision-making about one’s environment [301]. This behavior is crucial and, like other sensory systems and the cerebellum’s surrounding inhibition capabilities, engages in high tonic glomeruli activity while reserving other glomeruli for successive odor identification [301]. This type of “lateral inhibition” allows for increased contrast detection, a phenomenon found in many other cortical networks [302]. Yet, here, this activity may be restricted and linked to the animal’s movement, as head-restrained animals were absent in the study of the sniffing action in odor identification [301]. This point may be linked to the head-tilt positioning results found in drug application studies by [250] and offer corroboration that mechanoreceptive information from the nose, head, and neck are exceptionally vital and its integration swiftly and simultaneously is needed to ensure one’s survival. Nonetheless, airflow is known to increase across the olfactory epithelium during active sniffing and may highlight an undervalued role of the olfactory receptors to act as additional mechanoreceptors [303]; notably, all olfactory stimuli, whether mechanical or chemical, are directly transmitted to the olfactory cortex. Said plainly, one can almost immediately alter one’s neurological state by mechanically blowing air into one’s nostrils.

Additional support for the above hypothesis can be gleaned from the peripheral interface of the olfactory and trigeminal systems and the activation of overlapping cortical regions centrally [184,304,305,306]. Both sensory systems reciprocally augment and suppress each other yet retain specific roles for human function [304,307,308,309]. Other reports show that once engaged, the two systems result in cortical activation that is more significant than the two systems alone [310]. These results can explain the robust response cortically within one to two breaths of the EBM, as both CO_2_ and airflow activate both systems, albeit with different nasal, cranial nerve, and cortical biases. In general, the trigeminal system lateralizes or locates odorants and is responsible for processing sensory information like pungency, freshness, warmth, stinging, and burning, while the olfactory receptors have a bias toward odor detection [304,307,311]. Schaefer et al. report the presence of trigeminal collaterals arising from the nasal epithelium ethmoidal nerve and directly innervating the glomeruli of the olfactory bulb [312]. These fibers also appear to have centrally directed axons entering the TG and, eventually, the spinal trigeminal tract [312]. While there seems to be no additional direct evidence supporting these findings that we could find, it does give an anatomical substrate that would explain the direct peripheral interactions of the olfactory and trigeminal systems. A reason for the lack of corroboration is that there is much less known and understood about the human olfaction system than animals [313]. For a contemporary comparative assessment of the human olfactory system, see [313]. Clinically, this information is modern, as it relates to the possible non-invasive treatment of seizures and is also relevant in olfactory loss distinction in Parkinson’s disease [311]. See [311,314] for further discussion and application.

In addition to the clinical significance of mechanical airflow through the nose and subsequent trigeminal and olfactory stimulation [303], CO_2_, as described above, plays a critical role in human survival. There are central and peripheral chemoreceptor regions in humans. The peripheral sensors are located in the aortic and carotid bodies and respond to partial pressures of oxygen, carbon dioxide, and pH changes [315]. Please see [316] for review. In contrast, as described above, the central chemoreceptors laden throughout brainstem regions and cortical substrates, like astrocytes [316], are highly sensitive to pH and CO_2_ concentrations [130,315]. The central chemoreceptors within the brainstem promptly react to elevations in CO_2_ as hypercapnia is deleterious to human health and function. Consequently, two righting effects, known as the Bohr and Haldane effects, are known to occur. In the former, an increase in O_2_ hemoglobin affinity increases when CO_2_ is low, and in the latter, increased offloading of CO_2_ from the peripheral tissues occurs when O_2_ levels are above normal levels [315]. Also, when CO_2_ is above homeostatic levels, it drives O_2_ to dissociate from hemoglobin, resulting in higher serum O_2_ levels to maintain physiological equilibrium [121]. CO_2_ concentrations, whether systemically or cellularly, are adjusted and adapted constantly to ensure fundamental adaptive responses in line with the person’s acid–base (pH) balance.

There are three recognized ways that molecules entering the nose can reach the brain: extracellular diffusion, either by passive diffusion or convection; intraneuronal transport through the olfactory sensory nerves; or intraneuronal transport via the trigeminal nerve residing in the respiratory epithelium [317]. CO_2_ is a small lipophilic molecule transported across the blood–brain barrier through transcellular diffusion [318]. CO_2_’s molecular high permeability to the BBB indicates the importance and speed at which this chemical stimulus drives (respiratory) and alters neuronal excitability throughout the CNS. Minor deviations in pCO_2_ immediately cause changes in total minute ventilation [319], and lung studies have shown that CO_2_ affects regional perfusion and ventilation, while oxygen only acts on the latter [320]. Increases in CO_2_ levels will rise from increased metabolic and hypoventilation states (decreased central respiratory drive), like seizures [319]. However, surprisingly, humans can somewhat tolerate hypercapnia, possibly because of the body’s large tissue capacity for CO_2_ storage [319]. Therefore, when a person has a seizure while hypothetically shifting towards a hypercapnia state due to the high metabolic activity, diffusion of those levels via physiological storage mechanisms occurs. They remain potentially and relatively normocapnic or hypocapnic. Recall on a cellular level, extracellular acidosis, is brief during seizure activity (see CO_2_ and Chemosensitivity above and [55]), and it is the anticonvulsant effects of CO_2_ that are required to “reset” the neuronal pH and terminate seizure progression. There is also ample evidence, as described, that hypoventilatory states or altered gradients are expressed at varying degrees across PWE, and there is a loss of the reflexive central inspiratory drive that is usually initiated.

Yet, conversely, when a person intentionally holds their breath (transient apnea), there is a steep initial increase in CO_2_ concentrations of more than 3×, which occurs within seconds; this slows rapidly to a third of that if prolonged [319]. We suggest from these mechanisms and our literature review that the exhaled breath of the provider delivers concentrated levels of CO_2_ (up to 5×), “forced” airflow velocity, and enough of a relative temperature differential to “reset” the dynamic intracellular and extracellular hyperexcitability state throughout the seizures network involving several viable paths. Central to this idea is that respiratory CO_2_ levels appear to have a more robust triggering response than metabolic CO_2_ [129], which is credible, as mammals have co-evolved with Earth’s atmospheric domineering levels (96%) of CO_2_ to a mere 0.04% today [316]. For a historical timeline, see [316]. CO_2_’s potential effect on inherent human functions can be illustrated when the amygdala is lesioned bilaterally and exposed to severe CO_2_ concentrations (35%); fear will be aroused, yet this remains absent with environmental threats [176]. Conceivably, this can be explained because interoceptive threats, like high levels of CO_2_, are weighted with greater intensity than external environmental threats [321,322]. Understanding this, we can appreciate the development and difference of relative acute (fast) responses utilizing ion channels (along with other mechanisms described above) within peripheral and central chemosensors and chronic, slower-adapting gene expression and transcription factors in monitoring gas exchange between O_2_ and CO_2_. Reasonably, physiological CO_2_ chemosensitivity regulation will have different outcomes. In acute hypercapnia, the respiratory motor drive will increase, while in states of chronic hypercapnia, like COPD and other diseases, the respiratory motor drive will be depressed [316]. Adding to this dynamic is the interaction of the gas gradients between the person and the atmosphere that notably changes under increased metabolic oxidative phosphorylation activity [316], as expressed during seizures. The relative CO_2_ within the person compared to the atmosphere theoretically escalates; therefore, it would seem counterintuitive that applying nasal CO_2_ would be therapeutic.

However, as stated, our capacity to manage and disperse rising CO_2_ levels is reasonably efficient. We theorize this offsets the escalation, perhaps to the degree that perpetuates a more alkaline state and resultant neuronal hyperexcitability. If we view seizures as a global and neuronal “bioenergetic crisis,” then the sudden application of one person’s EB intranasally to a person amid a seizure is practically sound. To regulate and maintain all aspects of gas exchange and pH homeostasis, a triad of components interact and integrate dynamically and fluidly, like neural mechanisms, sensory and chemical activities, including metaboreceptor and chemoreceptor input systems, and mechanoreceptor and nociceptor facilitation [323]. Please review [323] for a pictorial demonstration. CO_2_ signaling is complex and still being explored. A complete elaboration is beyond this discussion, and we defer to articles by Cummins and Phelan [316,324].

We want to briefly address the plethora of research describing fear and panic in both humans and animals with exposure to high levels of CO_2_ [176,321,325,326]. CO_2_’s influence and resultant physiological and neuronal consequences are complex, and contextual application is necessary. Comparisons between rats and human studies investigating CO_2_ effects should also be made judiciously. Nevertheless, Amendola et al. summarize that “ventilatory and cardiovascular changes due to CO_2_ inhalation” run parallel with corollary emotional responses [176]. Additionally, it appears that a combinatorial threshold of CO_2_ tolerance is contextually determined. In general, rats can tolerate being exposed to lower concentrations of CO_2_ (<10%), meaning limbic-based behaviors are not aroused [176]. In this context, it is known that structural and physiological changes quickly occur due to seizures, hence expressing different phenotypical, physiological, and neuronal constructs that must be considered, yet toxicity reactions to CO_2_ have not been evaluated in seizure or epileptic models. There is early evidence in an animal model that one of the therapeutic mechanisms of action behind the ketogenic diet and reduction in seizures is increased duration and pacing of breathing, leading to decreased expiratory CO_2_, resulting in statistically significant intracerebral acidosis compared to the sham group [80].

Central to the altered behaviors mentioned is the amygdala, which notably contains ASIC1s profusely distributed throughout its neurons and astrocytes [80,326]. These channels are exquisitely sensitive to an acidic pH [327] and are sodium-selective ion channels [80]. TRPVs located in the PNS (intranasally) and ASICs located in the PNS and CNS function as highly tuned pH sensors that are activated by acidosis and promote neuronal activity, yet extracellular acidosis acts to also inhibit neuronal activity through inhibition of specific excitatory ion channels [327]. Acidosis causes activation of ASICs in the inhibitory neurons, which equals an endogenous counter-control to hyperexcitability [80]. During high neuronal activities, like seizures, local pH fluctuations rapidly occur and are affected and driven by complex mechanisms. See [55,149,327] for details. When this occurs, regional ionic subsystems are activated, which decreases neuronal activity, but countermeasures are, under normal circumstances, also implored to restore functional neuronal excitatory tone [149]. More recently, Alijevic et al. manipulated and observed time-dependent reactions of the ASICs, which were further tuned and codefined by local calcium, potassium, and proton concentrations [327,328]. A time-sensitive pH ramping can alter the intensity of response from the ASICs and, thus, action potential activity. In short, if the pH ramp ranges from 4 to 10 s, there is more significant neuronal firing than during a ramp of greater than 10 s [327]. Please see [327] for the specific mechanisms. Additionally, CO_2_ has a small kinetic diameter, and the surface diffusion rate is determined by the contact interaction between the “pore surface” and the gas itself, which is partially determined by its vapor pressure [329]. Although CO_2_ naturally has a higher vapor pressure due to its nonpolar structure, it is heavier than the air that carries it intranasally, thus establishing greater contact, partial condensation, and an increased potential diffusion rate [329]. The timing of the delivery of the EBM, along with its constituents, falls squarely within this time frame and physiological requirements to facilitate a time-dependent biological response in seizure termination through a faster pH change, causing sudden resetting of inherent mechanisms that control the overall tone of neuronal activation.

In PWE, autonomic dysfunction is known and documented, especially in longstanding epilepsy [157]. Sainju et al., evaluating respiratory variability in GCS, demonstrated in the first human study that postictal hypoxemia was common and concluded that central CO_2_ drive is needed to stabilize respiratory control postictally [157]. Furthermore, they confirmed Teran et al.’s findings of postictal transient chemosensory inhibition [155]. Like many human functions, there is a proposed range of respiratory variability. It is sufficiently documented that the postictal cortical breathing drive is abolished in many PWE [157]. Early interventions, such as body repositioning and supplemental oxygen, have been reported to halt this activity [157]. We extrapolate and propose from this that the early intervention of the EBM contributes to a significant arousal effect sufficient to retune and restore the chemical neural drive.

We speculate that a few scenarios may exist to explain the mechanistic plausibility of the EBM during seizures and account for the positive impact through several routes within a diverse group of PWE. The sudden concentrated yet restricted increase of CO_2_ through the EBM is immediately absorbed intranasally and disseminated to profuse areas in the cortex and brainstem, promptly influencing the local and global cellular pH and resulting in prompt seizure termination. As stated previously, pH’s magnitude and rate change are crucial factors in sensory nerve activation through various pathways [330]. Moreover, an immediate effect on pH is probable as CO_2_ itself is highly acidic at 3.7 at one atmosphere (National Center for Biotechnology Information (2024). PubChem Compound Summary for CID 280, Carbon Dioxide. Available online: https://pubchem.ncbi.nlm.nih.gov/compound/Carbon-Dioxide (accessed on 23 September 2024).

There is no doubt that CO_2_ is evolutionarily significant to humans and, therefore, has the probability to affect the epileptogenic network regardless of the central integrated state of the PWE. Trigeminal activation from the EBM can “reset” and stabilize neuronal excitability through the barrage of mechanical stimulation that reaches the spinal cord, thalamus, insular cortex, PAG, somatosensory cortex, and orbitofrontal cortex. Along with the mechanical activation, CO_2_ chemically activates nociceptive regions in the brainstem, the VLP thalamus, both primary and secondary somatosensory cortical structures, the insula, and the piriform cortex [185]. Likewise, in human studies, CO_2_ inhalation activates the amygdala, anterior insula, hypothalamus, and periaqueductal gray regions [176]. CO_2_ exposure will also show neuroendocrine and sympathetic activating responses [176]. The neuronal impulses induced by the intranasal airflow, CO_2_, and temperature receptors travel through multiple pathways simultaneously. From the nasal epithelium along the TGN and olfactory nerve, afferent information is delivered to the cortex and brainstem in streams. Analogous to dorsal and ventral visual streams that disseminate vast amounts of visual field information through cortical and brainstem structures determined not only topographically but also locationally and functionally to drive and manipulate behavioral reactions, the nasal information can be seen to travel anatomically superiorly and inferiorly in “streams”, depositing the relevant information to neuronal substrates along the paths. Moreover, as the TGN stimuli descend through the brainstem, they give off collaterals to the relevant respiratory network substrates before integrating into the dorsal cord and ascending contralaterally to the thalamus. Regarding the phenotypic patterns of hypoventilatory states postictally, the activation of the TRP cool thermoreceptors will directly stimulate respiratory centers [331].

There are neurological consequences of an acidic environment. The nasal trigeminal system strongly influences protective and reflexive behaviors, like breath rate, nasal cycle, naso-nasal and naso-ocular reflexes, naso-cardiovascular reflex, naso-respiratory reflex, and the foot cooling reflex [193]. In contrast, the olfactory system impacts digestive functions, memory, and social behaviors, synergistically working together to provide a vast repertoire of peripheral chemosensory and somatosensory information that will centrally inform, update, and dictate appropriate environmentally and biologically driven human behaviors [193]. The trigeminal free nerve endings nestled between the specialized chemosensory cells also contribute to sensory processing from local changes in temperature, pH, chemical (CO_2_), humidity, and pressure changes via specialized receptors [193]. Further contributing to behavioral responses is the autonomic nervous system, which contributes to glandular secretions, vessel diameter determinations, nasal congestion, and general patency of the nasal environment in reaction to the external world [332].

Perception of chemosensory information, conscious or unconscious, from the external environment begins with airflow through the nares. The nasal epithelial mucosa is not homogenized throughout; it displays increased chemosensory sensitivity due to a vast number of solitary chemoreceptor cells found in the anterior nasal region and increased mechanoreceptor capabilities sensitivity in the mid to posterior areas [333]. Consequently, blown air from one’s mouth to another’s nose as in the EBM, once passing through the internal nasal valve, travels across the nasal mucosa, stimulating cool receptors that respond from 0–37 degrees Celsius. A temperature gradient is created between the now cooling superficial mucosa and the warmer, deeper submucosa as evaporation and heat transfer occur; this effect is enhanced with higher flow rates and dryer and cooler air [334]. Under normal conditions, as cool, dry air passes through the nose, it reduces inspiratory muscle activity; this effect on ventilatory response also holds when combined with low concentrations of CO_2_ (2–6%); however, this reflex is absent and reversed when the air is humidified and contains higher CO_2_ levels, which results in lower nasal resistance and increased respiration [334]. This physiological model is germane, as many PWE suffer from uncorrected apneic states.

Similar to the mammalian dive reflex, a normal physiological response (see [335] for details), respiration is slowed because of the trigeminal activation synapsing in the nucleus solitarius’ ventrolateral subnucleus, located in the dorsal medulla, which houses primarily the inspiratory neurons of the dorsal respiratory group (DRG); these neurons have been shown to fire in phase with inspiration, yet seemingly independent of external afferent stimuli [336]. In animal models, the DRG neurons likely target cervical inspiratory neurons located in the spinal cord at C1–C2, and in turn, this region sends proprioceptive fibers to the nearby phrenic nucleus and thoracic motoneurons to coordinate inspiratory activity [337]. Concurrently, mechanical stimulation from the airflow will activate trigeminal nerve receptors and travel through the trigeminal ganglion down the brainstem and the trigeminal spinal tract while also sending off collaterals to synapses into the upper cervical cord, before ascending contralaterally to the VPM of the thalamus [294]. Note that these thresholds for trigeminally provoked apnea, olfactory identification, and discrimination are more significant in persons assigned female at birth [338].

The overall result is that nasal chemosensory and mechanosensory information are integrated to regulate breathing patterns [336]. Yet, they are not weighted equally. The trigeminal activity, responding to CO_2_ (chemical and nociceptive) and mechanical stimuli, responds more rapidly than the olfactory system and further increases its responsiveness with short-interval recurrent activation, guaranteeing a more robust central and temporal summation [332]. The trigeminal system also assures lateralization recognition of a presented nasal stimuli, and compared to odorless air, odorless CO_2_ concentrations activate not only the primary and secondary somatosensory regions, brainstem, and supplementary motor area, but also cortical olfactory regions [332,339].

The trigeminal system responds to mechanical stimuli, pain, and chemical stimuli via C and Aδ fibers, presumably from increased local proton concentrations (increased acidification) [338]. Additionally, the free trigeminal nerve endings are activated by water-soluble molecules, like CO_2_, above the tight junctions via the adjacent solitary chemosensory cells, yet also below the tight junctions by CO_2_ [332,340]. CO_2_ is not only water soluble but is likewise lipophilic and, therefore, able to penetrate the nasal mucosal tight junctions to directly activate the trigeminal free nerve endings, as shown by increased trigeminal event potentials even in the absence of mechanoreceptor activation [332]. Furthermore, when there is a drop in pH, the intranasal low pH-activated sodium ion channels (ASICs) located on the trigeminal nerve endings will be triggered, quickly leading to increased neuronal firing rates [341]. The sudden and brief pH shifts cause rapid configurational changes within the channels, representing closed, open, or desensitized functional status [341]. However, the channels are not only sensitive to detecting chemical shifts, but they also help form mechanosensitive complexes in neurons of the *C. elegans* and are thus capable of sensing light touch, therefore demonstrating the ASICs’ ability to act as pain receptors and mechanosensors; both integral to human functioning [341]. Genetic changes (SNPs) or disruptions in these ASICs are documented to increase seizure activity [341]. See [341] for details on ASICs’ molecular structure and function.

Also expressed on trigeminal nerve endings are the transient receptor potential (TRP) proteins. These cation molecular sensors conserved from the Drosophila to humans are a “superfamily” of polymodal sensors that detect an extensive range of stimuli, contributing to the human sensorium of taste, vision, hearing, touch, thermal detection, and proprioceptive experiences [197]. Like the ASICs mentioned above, TRPs also appear to have mechanosensitive abilities. Liu and Montell et al. suggest that the diverse sensor capabilities of the TRP channels are due to their activation by the underlying effects of mechanical force, whether the initial signaling cascade was mechanical or chemical. The generation of mechanical force is postulated as a unifying theory that dictates the TRPs’ architecture, thus explaining how these channels can respond to such diverse stimuli [197]. These channels appear activated by tension and distortion of the cell membrane in which they reside. Therefore, they are described as “stretch-activated and mechanosensitive channels” that allow us to perceive and “sample” not only the external world but also our internal world [197]. Further support for force activation is the speed at which the TRPs can respond to a given stimulus, such as touch, in adult Drosophila, occurring in 1–2 milliseconds [197]. The TRPA1 channels are quickly sensitized and activated by extracellular acidosis, chemical irritants, mechanical distortions, and thermal changes [342], and as such, are considered the facilitators of human behavior and evolution as they existed before the nervous system [200]. Please see [200,342] for a detailed review of TRPs’ history, evolution, and classifications.

In summary, TRPs and ASICs transduce chemical and sensory stimuli involving mechanical configurational changes. Located throughout the intranasal environment, both are activated by pH, chemical, thermal, and mechanical stimuli, resulting in increased neuronal firing. TRPV1 is expressed in sensory trigeminal nerves such as C fibers and Aδ and, once activated, such as in acidic environments, increases neuronal firing [343]. These channels are linked to long-standing pathological diseases like migraine, diabetes, and peripheral neuropathies. Yet, more vitally, they are responsible for defensive and protective behaviors that require immediate actions against deleterious ecological substances [344]. As a result, once activated, they can elicit fast-acting reflexes such as cough, decreased respiration, and bronchoconstriction [344]. We recognize an argument that the speed of conductance and influence of type C and Aδ fibers as compared to alpha motor neurons may not be substantial enough to influence global networks; however, we argue that these reflexes activated in response to environmental triggers and threats are the basis of fundamental evolutionary mechanisms that ensure survivability and, therefore, are neurologically weighted with greater importance. The significance of neurophysiological processes that occur with defensive responses is witnessed in adaptive behaviors such as “defensive immobility” due to self-protective actions [345]. de Mello Rosa and colleagues observed increased and prolonged respiratory rates in an animal model with activation of the ventrolateral PAG, an established hub for enhanced sensory processing to execute defensive actions [345]. While investigations are still being carried out to understand fully the complex neurological substrate involved in our survival, it is reasonably clear that autonomic activities, like respiration, in the face of an outward threat are immediately and centrally incorporated with motor behaviors to promote survival.

The brain’s ability to initiate and self-terminate seizures is multifactorial and occurs within pathways and physiological processes that can act in “tens of seconds” [328]. For abrupt termination, many proposed mechanisms are suggested: upregulation of sodium-potassium pumps, acidosis, thalamocortical long-range communications, increased neuronal synchrony, astrocytic activity [131], adenosine release, amplified inhibitory neuronal function, neurotransmitter depletion (glutamate), and ionic processes restored and maintained [149,328,346]. High neuronal activity occurs during seizures, causing rapid local pH fluctuations; these deviations are then affected and driven by complex mechanisms. See [55,149,327] for details. When this occurs, physiological systems are activated to restore function by decreasing neuronal activity, yet countermeasures to the restoration are also implored to restore functional neuronal excitatory tone [149]. The TRPVs located in the PNS (intranasal) and ASICs in the PNS and CNS function as highly tuned pH sensors and play an essential role in this paradigm. They are activated by acidic conditions and promote neuronal activity, yet extracellular acidosis inhibits neuronal activity by inhibiting specific excitatory ion channels [327]. Recently, Alijevic et al. manipulated and then observed time-dependent reactions of the ASICs and found that the ASICs are further tuned and codefined, not only by the pH but also by local calcium, potassium, and proton concentrations. Additionally, they found that time-sensitive pH ramping can alter the intensity of response from the ASICs. In short, if the pH ramp ranges from 4 to 10 s, there will be more significant neuronal firing than during a ramp of greater than 10 s [327]; this means a relatively shorter application of CO_2_ or pH change could have more immediate effects than if the change occurs slowly. Please see [327] for the specific mechanisms. We extrapolate that the exhaled breath applied during the EBM falls squarely within this time frame, and the sudden local pH changes that occur intranasally have the realistic probability of altering the CNS’s neuronal activity.

Even minor pH changes are biologically significant and, therefore, sensed and tightly controlled by almost all mammalian cells; this tight regulation occurs by altering the functionality (speed) of ion channels and transporters that shuttle the necessary acids or bases across the cell membrane to tightly maintain the local pH homeostasis [347]. These “acid extruders and acid loaders” respond to concentration changes in H^+^ in both peripheral and central chemoreceptors that directly adjust associated breathing mechanics and respiratory rates [324]. Two relevant proton detectors highly expressed in the retrotrapezoid nucleus (RTN) are TASK-2 and GPR4 [324]. These two proteins are essential in the central respiratory reflex arc from the RTN to the previously mentioned brainstem nuclei to control the respiratory muscles to preserve the desired pH. CO_2_ profoundly and fundamentally influences so many physiological processes in plants and animals that the survivability of many species would not be possible without it. The respective changes in the respiratory network’s pH are mainly because of CO_2_’s influence and role in cellular function and signal transduction throughout essential “core CO_2_ hubs” [324]. We encourage the reader to review reference [324] for an outline and review of CO_2_’s essential role in biological processes, signal transduction, human pathologies linked to CO_2_’s functionality, and its cellular cross-talk with other signaling pathways. CO_2_ uniquely affects biological systems, as the molecular gas itself controls certain cellular activities and indirectly modulates pH status, producing both CO_2_-dependent (increased concentration) and -independent (changes in the pH medium) pathways, thereby affecting essential biological events in complex ways.

However, we need to examine and appreciate the significance and influential role that mechanical and nociceptive information from the trigeminal system has on our neurobiological system. Respiration, trigeminal activity, and locomotor rhythms share coinciding brainstem and midbrain regions and are neurologically entrained for coordinated functionality [348]. For example, essential mammalian functions must be able to initiate effective and coordinated defensive behaviors when encountering environmental dangers. Mammalian survival depends on recognizing, processing, and responding to these stimuli with effective movement strategies. Many layered brain-wide circuits exist to subserve context-driven behaviors, and a full review is beyond this discussion. We refer the reader to [349] for a greater review. Yet, an example can be seen with olfactory and trigeminal information, cardiorespiratory execution, and locomotor functions that are interconnected through known neuronal matrixes and networks [348,349]. Orchestrating these exploratory or defensive movement strategies and actions requires input from the amygdala, hippocampus, hypothalamus, periaqueductal gray, mesencephalic and subthalamic locomotor regions [349].

Central to this arrangement is the PAG, which acts as a central hub receiving afferents from the prefrontal cortex, amygdala, hypothalamus, midbrain, brainstem, and spinal cord [350]. Cervical spinal cord and trigeminal afferents [186] also ascend to the PAG, converging with additional afferents from the NTS, CB, and the brainstem respiratory centers to affect the appropriate behavior [350]. Even movement of the mandible, a trigeminal activity, is entrained with phases of respiration, which are also associated with increased discharges from both peripheral and central chemoreceptors [348]. Equally, the spinal cord locomotor activity promotes trigeminal nerve discharges that synchronize respiration, jaw (mandibular) movements, and locomotion [348]. Yazawa et al. demonstrated the reciprocal functional interplay between the brainstem subsystems (respiratory rate), the trigeminal system, and mechanoreceptive and nociceptive input from the upper spinal cord during fictive locomotion during nasal and mouth breathing. They concluded that the reticulospinal pathway is the probable tract that transmits the added sympathetic tone needed for appropriate vascular control [348].

Another example of the complex connectivity involved in managing fundamental human functions, such as proper respiration, accurate neuronal activity, and precise movement patterns to ensure survivability, is the presence and recent appreciation of the cerebrospinal fluid-contacting nucleus (CSF-contacting nucleus). This nucleus lies within the ventral brainstem, bordering the inferior PAG and fourth ventricle [351]. It has “4 functional areas with 112 sub-regions of the brainstem and spinal cord” and is morphologically and physiologically similar to a cranial nerve [351]. The CSF-contacting nucleus is the proposed non-synaptic bridge between the CSF, nerves, glial cells, and blood vessels. It connects the spinal cord with the brainstem to manage pain responses, autonomic function, and arousal, thus involving PAG, reticular formation, the trigeminal nerve, and the NTS [351].

## 14. Limitations of Study

Our research team developed a comprehensive mixed-methods investigation protocol aimed at rigorously evaluating the Exchange Breathing Method (EBM). The design incorporated evidence-based practices for research methodology and validation, encompassing both human and animal models to explore physiological mechanisms and therapeutic outcomes. Despite the robustness of the proposed design, we were unable to enroll any participants, and the study was ultimately terminated.

While the preceding discussion provides a detailed overview, it captures only a portion of the extensive literature connecting respiratory, autonomic, and neurobiological pathways relevant to seizure activity. The current review is not exhaustive, and further research is needed to fully elucidate the mechanisms and clinical applicability of the EBM within the broader context of epilepsy treatment.

## 15. Conclusions

This review has outlined the neurobiological pathways implicated in seizure disorders and evaluated historical and current treatment modalities in light of evolving clinical understanding. The physiological mechanisms discussed, particularly those governing autonomic and respiratory regulation, are deeply conserved and likely serve adaptive roles in maintaining neural homeostasis. We propose that the Exchange Breathing Method (EBM) operates as a robust combinatorial input, engaging multiple physiological systems simultaneously to disrupt seizure activity in real time.

There exists a compelling physiological rationale to support anecdotal reports of seizure attenuation following the directed exhalation of air into a subject’s nostrils. The mechanism may involve acute modulation of autonomic nervous system tone, brainstem reflex arcs, or oxygen/CO_2_-sensitive networks. These systems are functionally interlinked, suggesting that even brief respiratory interventions may exert a significant neuromodulatory effect. To validate the therapeutic potential of the EBM, there is a pressing need for rigorously designed, mixed-methodological studies, including controlled human trials and translational animal models, that can quantify its impact on seizure onset, duration, and recurrence. Such investigations may uncover novel pathways for non-invasive seizure intervention and broaden the landscape of epilepsy treatment options.

Table 1 (epilepsy treatment methods) compares different epilepsy treatment modalities, including pharmacological, surgical, and novel non-invasive interventions, highlighting mechanisms, advantages, and limitations of each method.

Table 2 (ASIC vs. TRP channels) provides a comparison between acid-sensing ion channels (ASIC) and transient receptor potential (TRP) channels regarding their distribution, stimuli responsiveness, physiological roles, and potential seizure modulation properties.

## Figures and Tables

**Table 1:** Epilepsy treatment methods.

Treatment Method	Mechanism of Action	Advantages	Limitations
Antiseizure Medications (ASMs)	Target neuronal excitability, ion channels, neurotransmitter modulation	Readily available, widely studied, non-invasive	1/3 of PWEs are non-responsive; side effects
Surgical Resection (e.g., for TLE)	Removal of epileptogenic tissue	Effective for drug-resistant focal epilepsy	40% seizure recurrence at two years; invasive
Intranasal Drug Delivery	Direct delivery of drugs via nasal cavity to brain (e.g., benzodiazepines)	Bypasses blood–brain barrier; rapid effect	Requires formulation; not suitable for all drugs
Olfactory/Trigeminal Stimulation	Stimulation of nasal chemosensory/trigeminal pathways to inhibit seizures	Non-pharmacological, may abort seizures rapidly	Limited empirical validation; variability in stimuli
Exchange Breathing Method (EBM)	Airflow and CO_2_-triggered stimulation of TRP and ASIC channels	Safe, non-invasive, fast acting, targets multiple mechanisms	Requires partner assistance; early research stage

**Table 2:** ASIC vs. TRP channels.

Characteristic	ASIC (Acid-Sensing Ion Channels)	TRP (Transient Receptor Potential) Channels
Location	CNS and PNS (esp. amygdala, trigeminal endings)	Widely distributed in PNS (esp. nasal epithelium)
Stimuli	Low pH (acidic environments)	Chemical, mechanical, thermal (e.g., CO_2_, cold, pressure)
Ion Selectivity	Na+ selective	Non-selective cation channels
Response Time	4–10 s ramp = high firing; >10 s = low firing	1–2 ms (fast response)
Role in Seizures	Activated during extracellular acidosis to inhibit hyperexcitability	Initiates protective reflexes (e.g., reduced respiration)
Effectiveness of Stimuli	Highly sensitive to rapid pH drops (e.g., CO_2_ inhalation)	Activated by CO_2_, airflow, cold, and pain
Dual Functionality	pH sensors and mechanosensors	Polymodal sensors with mechanotransduction

## Data Availability

Not applicable.
